# Palladium catalyzed radical relay for the oxidative cross-coupling of quinolines

**DOI:** 10.1038/s41467-022-31967-0

**Published:** 2022-07-19

**Authors:** Xiaorui Zhao, Xiaojuan Zhu, Kang Wang, Junqian Lv, Shangjun Chen, Guohua Yao, Junyu Lang, Fei Lv, Yinghui Pu, Ruoou Yang, Bingsen Zhang, Zheng Jiang, Ying Wan

**Affiliations:** 1 The Education Ministry Key Laboratory of Resource Chemistry, Joint International Research Laboratory of Resource Chemistry of Ministry of Education, Shanghai Key Laboratory of Rare Earth Functional Materials, and Shanghai Frontiers Science Center of Biomimetic Catalysis, Shanghai Normal University, Shanghai, P. R. China; 2 School of Chemistry and Chemical Engineering, Taishan University, Shandong, P. R. China; 3 School of Physical Science and Technology, Shanghai Tech University, Shanghai, P. R. China; 4 Shenyang National Laboratory for Materials Science, Institute of Metal Research, Chinese Academy of Sciences, Shenyang, P. R. China; 5 State Key Laboratory of Materials Processing and Die & Mould Technology, School of Materials Science and Engineering, Huazhong University of Science and Technology, Hubei, P. R. China; 6 Shanghai Synchrotron Radiation Facility, Zhangjiang National Lab, Shanghai Advanced Research Institute, Chinese Academy of Sciences, Shanghai, P. R. China

**Keywords:** Heterogeneous catalysis, Catalytic mechanisms, Catalyst synthesis

## Abstract

Traditional approaches for transition-metal catalyzed oxidative cross-coupling reactions rely on *sp*^2^-hybridized starting materials, such as aryl halides, and more specifically, homogeneous catalysts. We report a heterogeneous Pd-catalyzed radical relay method for the conversion of a heteroarene C(*sp*^3^)–H bond into ethers. Pd nanoparticles are supported on an ordered mesoporous composite which, when compared with microporous activated carbons, greatly increases the Pd *d* charge because of their strong interaction with N-doped anatase nanocrystals. Mechanistic studies provide evidence that electron-deficient Pd with Pd–O/N coordinations efficiently catalyzes the radical relay reaction to release diffusible methoxyl radicals, and highlight the difference between this surface reaction and C–H oxidation mediated by homogeneous catalysts that operate with cyclopalladated intermediates. The reactions proceed efficiently with a turn-over frequency of 84 h^−1^ and high selectivity toward ethers of >99%. Negligible Pd leaching and activity loss are observed after 7 catalytic runs.

## Abstract (web summary)

Traditional approaches for transition-metal catalyzed oxidative cross-coupling reactions rely on *sp*^2^-hybridized starting materials. Here the authors report a heterogeneous Pd-catalyzed radical relay method for the conversion of a heteroarene C(*sp*^3^)–H bond into ethers.

## Introduction

Transition-metal catalyzed cross-dehydrogenative C–O coupling reactions of the C–H bond have emerged as an increasingly powerful platform for the production of ethers and esters, because the use of C–H bonds as functional groups similar to carbon–halogen bonds is a valuable, straightforward, and atom-economical strategy for the construction of complex molecules^1–4^. Compared with the extensive studies on C–C coupling which have aimed at both understanding the detailed mechanism of C–H functionalization and designing useful functionalization schemes, research on C–O bond formation-based metal-catalyzed C–H bond activation is not as advanced, possibly due to the electronegativity of O as well as its strong coordination with metals that inhibit the effective reductive elimination^5,6^. In addition, C(*sp*^3^)–H bonds, in contrast to C(*sp*^2^)–H, have no empty low-energy orbitals or filled high-energy orbitals that readily interact with the orbitals of the metal center, as is the case with the π-groups^7,8^. Therefore, catalytic C(*sp*^3^)–H oxidative functionalization seems to represent a significantly bigger challenge^7,9,10^.

Pyridine derivatives have always attracted significant attention because of their diverse chemical properties and range of biological activity^11,12^. For example, 8-(methoxymethyl) quinolines containing additional C–O bonds, and their derivatives, have found many uses, including anti-inflammatory, antibacterial, and antifungal activity^11–13^. Notably, most research focuses on homogenous catalysis^5,6,14,15^. For example, Sanford et al. explored C–H bond acetoxylation and alkoxylation caused by pyridine derivatives using the homogenous catalyst Pd(OAc)_2_^16,17^. The catalytic cycle was proposed to involve ligand-directed C–H activation to generate a cyclopalladated intermediate which is the rate-determining step, two-electron oxidation of the palladacycle to generate a Pd^IV^ species via dimeric Pd^III^–Pd^III^ intermediates, and C–O bond-forming reductive elimination to release the product^18,19^. Heterogeneous catalysts have the advantage of catalyst recycling and can be used many times, as well as reducing or eliminating palladium contamination in the products. However, it is generally admitted that metal leaching into solution from immobilized metal complexes or supported metal particle precatalysts is inevitable as long as the coupling reactions occur on single metal centers following a M^0^/M^II^ or M^II^/M^IV^ catalytic cycle^20^. The oxidative addition of the substrate to support Pd^0^ and create soluble Pd^II^ species is key to Pd leaching for Pd/C and Pd^II^-exchanged graphite oxide^21^. In this case, palladium contamination in the products is still the restriction for the practical use of the method in pharmaceutical production^22^. Leaching, recrystallization or poisoning of the metal is very serious when pyridine derivatives are involved because they are strongly adsorbed on transition metal surfaces by the lone pair electrons^23,24^. Therefore, the C(*sp*^3^)–H direct oxidative functionalization of quinolines over a leaching- and aggregation-free heterogeneous catalyst is highly attractive, but has had limited research.

Nature often exploits the selective oxidation of C–H bonds by enzymatic systems, with the reactive metal–oxo species removing an H atom from a C–H bond, to produce a carbon-centered radical and an oxygen-centered radical species^25,26^. A homogeneous Cu-catalyzed carbon-centered radical relay strategy for C(*sp*^3^)–H cyanation and oxidation has been reported to form C–N and C–O bonds, which resembles nickel- and copper-catalyzed cross-coupling involving the reaction of a carbon-centered radical with a transition-metal center^25,27,28^. A radical removes a hydrogen atom from an *sp*^3^-hybridized carbon atom producing a benzylic radical that generates a functionalized *sp*^3^ center and undergoes C(*sp*^3^)–N/O bond formation^25,27^. Recently, we reported that heterogeneous Pd clusters can break the barrier to electron transfer in traditional homogenous catalysis and catalyze a unique bisarylation transformation (Supplementary Fig. 1)^29^. The parallel adsorption of thiophene over Pd clusters induces the simultaneous activation of the target C2(*sp*^2^)–H and C5(*sp*^2^)–H in thiophene, and subsequent bisarylation by aryl radicals on the surface. This strategy is distinct from the homogenous ones, and may improve the catalytic performance, as well as avoid the oxidation of Pd^0^ or Pd^II^ and leaching. These reaction paths remind us to consider how a radical relay reaction can be applied to C(*sp*^3^)–H oxidation over heterogeneous Pd catalysts. The key issues are (a) the adsorption of heterocyclic molecules with the activation of the target C–H bond to serve as the functionalized *sp*^3^ center with a moderate adsorption energy, and (b) the generation of oxygen-centered radical species which attack the “activated” C(*sp*^3^)–H bond resulting in the formation of C–O bonds.

Here we postulate that a heterogeneous Pd-catalyzed oxygen-centered radical relay strategy can provide the basis for the oxidative cross-coupling of C(*sp*^3^)–H bonds and alcohols using a heteroarene substrate. Pd nanoparticles are supported on an ordered mesoporous N-doped anatase-carbon composite. Compared with the frequently-used microporous activated carbons, the composite increases the Pd *d* charge by strong interaction with N-doped anatase nanocrystals and in turn changes the adsorption configuration of the reactant. Simultaneously, the ordered mesoporous solid reduces the mass transfer limitation, especially, for reactions involving large-molecules. The oxidative cross-coupling of the C(*sp*^3^)–H bonds of the quinolines and methanol over the heterogeneous Pd nanocatalysts is based on the surface adsorption-induced radical relay reaction, different from the mechanisms involving traditional homogeneous cyclopalladated intermediates and Pd^0^/Pd^II^ or Pd^II^/Pd^IV^ cycles. This surface adsorption-induced radical relay concept paves the way for the formation of diverse organic molecules and should find widespread use in organic synthesis, particularly for medicinal chemistry applications.

## Results

### Structural properties of Pd nanocatalysts supported on ordered mesoporous N-doped TiO_2_-carbon composites

An ordered mesoporous N-doped TiO_2_-carbon composite ((N)TiO_2_-OMC) was chosen as the carrier for the Pd nanoparticles, taking advantage of the strong metal-support interaction (SMSI) between Pd and N-doped anatase nanoparticles^30^, and the absence of mass transfer in the mesoporous composite^31^. The channels in the mesoporous composite were formed from small discrete TiO_2_ nanoparticles and carbon, and the mass transfer process was facilitated by these porous channel walls. The composite carriers were prepared using a self-assembly approach previously reported by our group, involving a sol-gel process for the production of a titanium sol in the presence of titanium chloride and urea, the co-assembly of a triblock copolymer, resol, and the titanium sol, polymerization, and carbonization^32^. A simple wet impregnation method was used to load the Pd nanoparticles. All of the raw materials are commercially available and all of the processes have been realized on an industrial scale^33^.

**Fig. 1:**
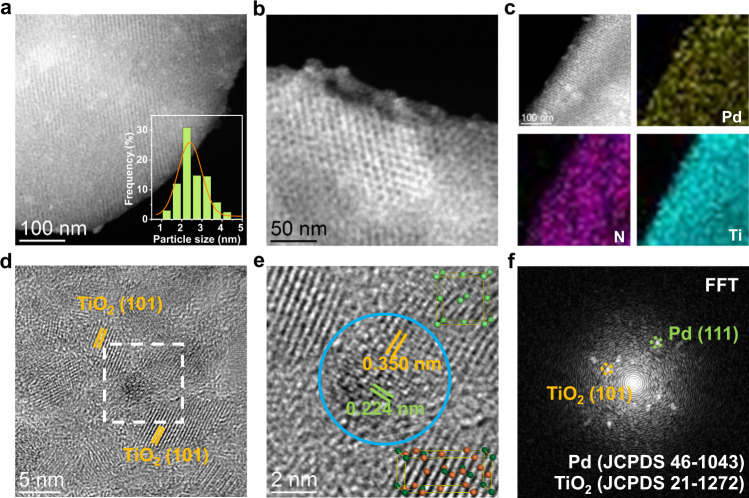
Structure of Pd nanocatalysts. **a, b** Representative HAADF-STEM images for Pd/(N)TiO_2_-OMC and **c** the corresponding Pd, N and Ti elemental maps. The inset in **a** is the Pd particle size distribution which was determined from at least 200 nanoparticles. **d** HRTEM image of Pd/(N)TiO_2_-OMC. **e** Enlarged view of the white square in **d**. The top-right and bottom-right insets correspond to Pd and TiO_2_ unit cells, respectively. **f** Selected-area FFT patterns of Pd and TiO_2_ from the area in the blue circle in **e**.

A scanning electron microscopy (SEM) image of the Pd/(N)TiO_2_-OMC catalyst shows typical bulk amorphous carbon with no large crystals and elemental maps show uniform composition distributions (Supplementary Fig. 2). Ordered, open mesopore arrays are seen in high-resolution SEM (HRSEM) images in large domains, and no large crystals on the outer surface are seen, indicating that the Pd nanoparticles, if any, are confined inside the mesostructure rather than growing outside the pores (Supplementary Fig. 3). High-angle annular dark-field scanning transmission electron microscopy (HAADF-STEM) images for Pd/(N)TiO_2_-OMC show highly ordered two-dimensional hexagonal mesopores and well-dispersed Pd nanoparticles with an average diameter of 2.3 nm (Fig. 1a, b). The size of the Pd nanoparticles was estimated from the exposed surface area measured by CO chemisorption to be about 2.2 nm, in good agreement with the TEM results. A HRTEM image of the composite carrier shows that the pore walls are constructed of nanocrystals and amorphous material (Supplementary Fig. 4 and Table 1), similar to our previous reports for an ordered mesoporous titania-carbon composite^32^. After loading the Pd, the structure remained the same, and the uniform distribution of Pd, N, and Ti in the solid was confirmed by STEM and EDS mapping (Fig. 1c and Supplementary Fig. 2). The Pd signal overlaps signals from N and anatase. The anatase nanocrystals were measured to be ~3.5 nm in diameter and are randomly oriented in the amorphous carbon pore walls (Supplementary Fig. 4). They have a lattice spacing of 0.350 nm which can be indexed as the (101) plane (Fig. 1d, e). The stable two-phase mesostructure has an amorphous carbon matrix containing N-doped anatase nanocrystals^32,34^. The interface between the Pd nanoparticles and the TiO_2_ nanoparticles can be clearly seen in the HRTEM images of the supported Pd catalyst (Fig. 1d, e). Fast Fourier transform (FFT) patterns of a selected area clearly give two lattice spacings of 0.350 and 0.224 (or 0.194) nm corresponding to the (101) plane for anatase and (111) (or (200)) for Pd (Fig. 1e, f and Supplementary Fig. 5). As a result, the Pd nanoparticles are mainly anchored on the surface of the anatase nanocrystals possibly due to the strong metal-support interaction between them, and the hydrophobicity of amorphous carbon^35^. The wide-angle X-ray diffraction (XRD) patterns for Pd/(N)TiO_2_-OMC show several diffuse peaks (Supplementary Fig. 6) which can be matched to anatase^32,36^, implying highly dispersed crystalline nanoparticles. The broad (002) peak of the carbon overlaps the anatase peaks^36^, but using the Scherrer equation for the anatase (101) reflection, the size of the nanocrystalline TiO_2_ particles is estimated to be 3.8 nm, in good agreement with the TEM results^36^. However, there is a weak diffraction peak at ~40.1°, which is attributed to very small Pd nanoparticles^29,37^. The anatase TiO_2_ content of the catalyst was estimated to be approximately 59 wt% by thermal gravimetric analysis (TG) (Supplementary Fig. 7), the nitrogen content was estimated by elemental analysis (EA) to be 1.27 wt%, and inductively coupled plasma-atomic emission spectrometry (ICP-AES) analysis showed 0.99 wt% Pd (Supplementary Table 1).

Type-IV N_2_ sorption isotherms with a sharp capillary condensation step at middle relative pressures were detected for the supported Pd catalyst, similar to the isotherms of the pristine mesoporous composite carrier, suggesting the good uniformity of the mesopores (Supplementary Fig. 8). The sorption hysteresis loops for both the carrier and the supported catalyst are of the H2 type, suggesting roughly cylindrical pores, possibly resulting from the different shrinkages of titania and the phenolic resin^34,36^. This result is the same as that for mesoporous inorganic-carbonaceous solids, implying non-uniformity of some local components^34^. The pore size was calculated to be 4.3 nm, and the width at half maximum of the pore size distribution was 1.8 nm, indicating a very narrow pore size distribution. The Brunauer-Emmett-Teller (BET) specific surface area and pore were calculated to be 402 m^2^ g^−1^ and 0.29 cm^3^ g^−1^, respectively (Supplementary Table 1). These results show that the Pd/(N)TiO_2_-OMC catalyst has open, ordered mesopores with no pore blockage.

For comparison, supported catalysts including Pd/TiO_2_-OMC, Pd/SBA-15, and commercial Pd/C were also used. The Pd nanoparticles were loaded inside the pores of a porous anatase/carbon composite, silica and carbon, with Pd particle diameter modal values of 2.5, 3.1, and 3.9 nm (Supplementary Fig. 9 and Table 1), respectively. A moderately high BET surface area (>350 m^2^ g^−1^), and pore volume (>0.25 cm^3^ g^−1^) were detected for all these catalysts (Supplementary Table 1). Pd/TiO_2_-OMC and Pd/SBA-15 have uniform mesopores with a range of pores sizes with modal values of 4.4 and 9.0 nm, respectively, and Pd/C has random mesopores (Supplementary Fig. 9).

### Electronic properties

**Fig. 2:**
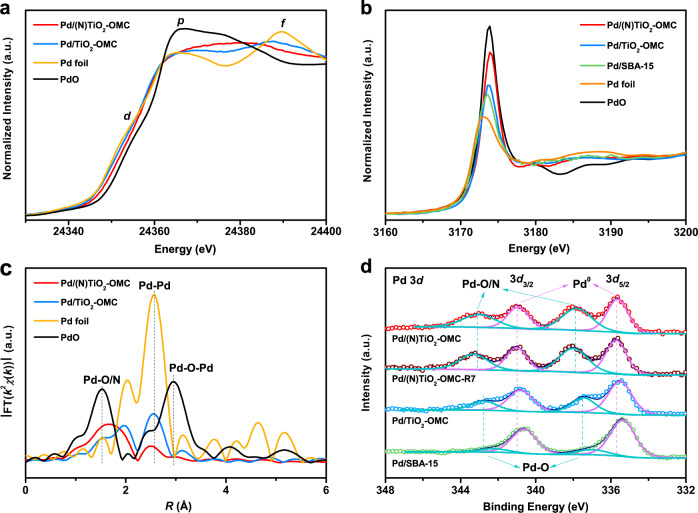
Electronic properties. **a**, **b** XANES spectra of the **a** Pd *K*-edge and **b** Pd *L*_3_-edge, **c** *k*^2^-weighted and FT-EXAFS spectra of the Pd *K*-edge, and **d** XPS spectra of the 3*d* level of Pd for Pd/TiO_2_-OMC and Pd/(N)TiO_2_-OMC catalysts. The reference samples include a Pd foil, PdO, and Pd/SBA-15. The Fourier transforms in **c** were not corrected for phase shifts. The Pd binding energies in **d** were fitted by peak fitting programs. Pd/(N)TiO_2_-OMC-R7 is the catalyst after being used in seven runs.

The Pd *K*-edge X-ray absorption near edge structure spectra (XANES) are shown in Fig. 2a. There are several features for a Pd foil, that is three peaks named *d*, *p*, and *f* arising from the states having 4*d*-, 5*p*-, and 4*f*-orbital characters, respectively, as well as two valleys which are caused by hybridization of the 5*p* orbitals with the 4*d* and 4 *f* orbitals. It is clear that both Pd/TiO_2_-OMC and Pd/(N)TiO_2_-OMC have a pre-edge similar to Pd foil, suggesting the metallic state of the Pd nanoparticles^29^. The pre-edge shifts to a higher energy and the *d* peak disappears for Pd/TiO_2_-OMC and Pd/(N)TiO_2_-OMC, indicating overlapping of the 4*d* band with the *s*(*p*) orbitals of the coordinated atoms^38^. The *f* peak of the studied catalysts shifts to lower energies with a large decrease in intensity, and the *pf* valley almost vanishes for Pd/(N)TiO_2_-OMC, which is ascribed to increased band hybridization^39^. The threshold region of the *L*_3_-edge XANES spectra of the Pd samples is used to calculate the unoccupied density of states of the Pd *d*-band above the Fermi level which is associated with the white line intensity (2*p*_3/2_-to-4*d* transition, Fig. 2b)^40,41^. It has been reported the small Pd particles are electron deficient^42^, so the Pd/SBA-15 catalyst, which has a similar Pd particle size to the studied composite-supported catalyst but a very weak metal-support interaction, is measured as a reference. The higher intensity of the white line for Pd/(N)TiO_2_-OMC relative to Pd/SBA-15 indicates a *d*-charge depletion at Pd sites in the nanoparticles^43,44^. A more effective hybridization between the *d* and *s*, *p* orbitals of Pd would therefore produce a positive threshold shift, in good agreement with the *K*-edge spectra. In fact, an isolated single-atom Pd coordinated with O or N has an increased white line intensity close to that of PdO, possibly due to losing electrons to the more electronegative O/N atoms^45^. In contrast, the white line intensity for Pd/TiO_2_-OMC is similar to that of Pd/SBA-15 with only a slight increase. The change in *d*-electron count is thought to result from (*s*,*p*)-*d* rehybridization or intra-atomic charge transfer^46^. Therefore, these phenomena indicate that the N species play an important role in changing the electronic properties of electron-deficient Pd. The respective estimated *d*-electron depletions are 0.03 e and 0.14 e for Pd/TiO_2_-OMC and Pd/(N)TiO_2_-OMC compared to the reference Pd/SBA-15^47^.

Typical *k*^2^-weighted and Fourier-transform extended X-ray absorption fine structure (EXAFS) spectra in the *K*-edge of Pd were obtained to analyze the effect of the orbitals in N-doped anatase on those in Pd and the bonding between them (Fig. 2c and Supplementary Fig. 10, Table 2). The characteristic features of Pd–O–Pd coordination in PdO are insignificant in the spectra of both Pd/(N)TiO_2_-OMC and Pd/TiO_2_-OMC. This phenomenon, together with the not obvious pre-edge feature characteristic of PdO for these catalysts in the XANES spectra, indicates that the atomic composition of the Pd catalysts is that of the Pd metal. The Pd–Pd coordination number (CN) for Pd/TiO_2_-OMC is reduced to 6.5 compared to 12.0 for a Pd foil, which is mainly due to the small Pd particle size in which many atoms are located at the surface^29^. The CN values for Pd–Ti with Pd located on top of an oxygen vacancy (O_v_)^48,49^, and Pd–O–Ti, are estimated to be 1.8 and 0.8, respectively. In contrast, the CN_Pd–Pd_ for Pd/(N)TiO_2_-OMC is suddenly reduced to 1.8. Taking into consideration the similar Pd particle size to that in Pd/TiO_2_-OMC, there is obviously a strong interaction between the N-containing anatase and Pd. Indeed, the CN value for Pd–O/N is estimated to be 3.8. The significant decrease in the CN value for Pd–Pd and the increase in CN for Pd–O have also been observed in the Pd/CeO_2_ catalyst caused by the SMSI effect (similar pre-edge shift in the *K*-edge XANES spectrum to this study)^50^. A very low CN value for Pd–Ti suggests that few Pd atoms are located at oxygen vacancies in Pd/(N)TiO_2_-OMC.

X-ray photoelectron spectroscopy (XPS) analysis was performed to investigate the oxidation state. The typical doublets for the Pd 3*d* XPS spectrum can be deconvoluted (Fig. 2d), and the major peaks for Pd^0^ and the minor peaks for Pd–O/N can be fitted for the studied catalysts. Compared with Pd/SBA-15 which has a similar particle size but a very weak interaction with Pd, the Pd 3*d* core levels of the Pd/(N)TiO_2_-OMC show a shift to a higher binding energy but for Pd/TiO_2_-OMC remains almost unchanged, indicating electron-deficient Pd due to the presence of N. It has been reported that an initial effect caused by differences in the Fermi level of the metal nanoparticle and the support tends to shift the binding energy of Pd on anatase to a lower value^49,51^, while the interaction between Pd and O due to the formation of a Pd–O–Ti bond results in an oxidation sate between metallic Pd and PdO which is determined by the particle size effect^52^. When the anatase carrier contains O_v_ and dopant atoms, the hybridization of the cluster changes due to a change in the work function^53^. Pd/TiO_2_-OMC contains O_v_, as indicated by electron paramagnetic resonance (EPR) spectroscopy (Supplementary Fig. 11). The Pd clusters prefer to be located on top of O_v_ in the absence of dopant atoms^49,54^. They are more negatively charged because O_v_ acts as an electron donor and increases the electron density on the cluster, as well as producing lower effective ionization potentials^49,54^. In this case, the TiO_2_ carrier, Pd–O–Ti, and Pd–O_v_ interactions are possibly neutralized compared to Pd/SBA-15, causing no obvious change in the Pd core-level shift. In contrast, a N–Ti–O linkage in the N-doped anatase carrier can be strongly bonded to Pd, possibly due to the efficient Pd−N coordination^55,56^. The Pd–O_v_ interactions are weak and electron transfer occurs from Pd to N because of the electronegativity difference. As a result, electron-deficient Pd is present on only the N-containing composite support. These phenomena are in good agreement with the EXAFS and XANES results (Fig. 2a and Supplementary Fig. 10).

### Direct oxidative coupling over Pd nanocatalysts

The direct oxidative coupling of 8-methylquinoline (8-MeQ, **1a**) with methanol (MeOH) using iodobenzene diacetate (PhI(OAc)_2_) as the oxidant was studied under mild conditions using a Pd catalyst supported on an ordered mesoporous composite. No other additives were used. Preliminary experiments were performed to exclude diffusion mass transfer limitations. The results showed that a stirring speed of 800 rpm in the batch reaction enabled the reaction to occur without diffusion limitations (Supplementary Fig. 12a). A Madon–Boudart test was performed to study the limitations of mass transfer^29^. The mentioned nanocatalysts were synthesized with similar particle sizes (2.2 nm) but different Pd concentrations (0.5–2 wt%, Supplementary Fig. 13 and Table 1). We proved that the initial reaction rate (*r*_0_) increases linearly with the concentration of palladium in the catalyst (Supplementary Fig. 12b). Mass transfer can be excluded possibly due to the fact that large and open mesopores with no pore blockages provide channels and space for reactant transport and all active Pd sites are exposed to the reactants.

**Fig. 3:**
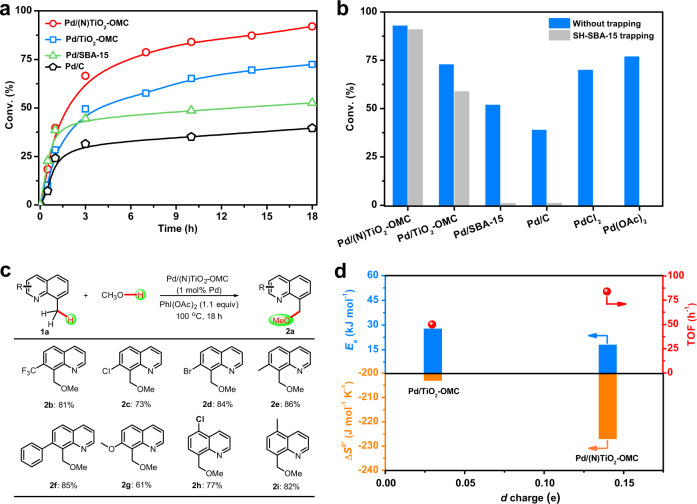
Kinetics study and substrate scope. **a** Conversion plots for the Pd-catalyzed direct methoxylation of 8-MeQ with reaction time. **b** Comparison of the conversion of 8-MeQ with and without the addition of a solid SH-SBA-15 trapping agent over the various Pd catalysts (S:Pd = 35 in molar ratio). **c** Substrate scope of the C(*sp*^3^)–H direct oxidative functionalization of quinolines catalyzed by Pd/(N)TiO_2_-OMC. **d** Relationship between the *d-*charge depletion at Pd sites and activation energy (*E*_a_, blue bar), TOF (red sphere), and the activation entropy (Δ*S*^0*^, orange bar) for the direct methoxylation of 8-MeQ over the Pd catalysts supported on TiO_2_-OMC and (N)TiO_2_-OMC carriers. Reaction conditions: 0.2 mmol of 8-MeQ; 0.22 mmol of PhI(OAc)_2_; 1 mol% Pd catalyst; 2 mL of MeOH; 100°C; 800 rpm; atmospheric pressure; in air.

The Pd/(N)TiO_2_-OMC catalyst has a high activity (TOF value of 84 h^−1^) and selectivity (>99%) in the methoxylation of 8-MeQ (Fig. 3a). The yield of 8-(methoxymethyl)quinoline (**2a**) reached 92%, higher than the reported homogeneous reaction and our result using Pd(OAc)_2_ as the catalyst (77%)^17^, and close to that for the multiwalled carbon nanotube supported Pd^II^ catalyst (99% yield with an estimated TOF of 99 h^–1^) by the Pd^II^/Pd^IV^ catalytic cycle^57^. In comparison, the TOF value for the N-free catalyst Pd/TiO_2_-OMC was reduced to 55 h^−1^, and the conversion of **1a** decreased to 72%. Pd/SBA-15 gives a rapid initial conversion with a similarly high reaction rate to Pd/(N)TiO_2_-OMC, but it undergoes a rapid deactivation after 1 h. The product yield is moderate (53%). Similar phenomena have been observed for commercial Pd/C.

We first attempt to exclude homogenous catalysis. Here a solid-trapping test was used to evaluate whether the soluble Pd species are involved in the reaction (Fig. 3b). A thiol-group-modified mesoporous silica (SH-SBA-15) was added to the reaction flask with a S:Pd molar ratio of 35 to trap any soluble palladium released from the catalyst, and therefore quench the homogenous reaction^37^. Indeed, SH-SBA-15 can quench a reaction catalyzed by either Pd(OAc)_2_ or PdCl_2_. There was no significant difference in the conversion of **1a** over Pd/(N)TiO_2_-OMC in the presence of SH-SBA-15, confirming negligible Pd leaching into solution (Fig. 3b). However, the reaction cannot proceed in the presence of SH-SBA-15 catalyzed by Pd/SBA-15 or Pd/C. Therefore, both Pd/SBA-15 and Pd/C serve as a precatalyst which leaches active soluble Pd species at the beginning of the reaction, as previously reported and results in a high initial reaction rate^29^. But the soluble species may undergo aggregation during the reaction to form inactive Pd black, resulting in a loss of activity^29^. In fact, the ICP-AES analysis shows approximately 20% leaching of Pd in solution after the reaction and an additional 20% leaching in the second run with a decreased yield of 21%, and the TEM images of the used catalyst show the aggregation of metal particles (Supplementary Table 3 and Fig. 14). The situation for the N-free catalyst Pd/TiO_2_-OMC was different, with SH-SBA-15 quenching only about 10% of the activity. This result may imply that ~10% Pd leaches from the solid catalyst. If the contribution to the activity by the leached Pd (estimated from the performance for Pd/SBA-15) is subtracted, the contribution of the heterogeneous Pd to the initial reaction rate is reduced to 24 mmol mmol_Pd_^−1^ h^−1^ (TOF value of 50 h^−1^). The avoidance of Pd leaching in Pd/(N)TiO_2_-OMC was confirmed by hot filtration, recycling tests and ICP-AES analysis. No further conversion of the reactant was observed in the filtrate after the hot infiltration of the solid catalyst (Supplementary Fig. 15). Both the TOF value which is dominated by the number of overall active sites and the conversion of 8-MeQ remains almost constant during 7 successive runs (Supplementary Fig. 16). After each run, the filtrate was collected and examined by ICP-AES, and the concentration of metal ions was below the detection limit (Supplementary Table 3). These results confirm that there is no leaching of Pd into the solution. After 7 catalysis runs, the catalyst structure did not change significantly, as shown by XRD patterns and TEM images, confirming no aggregation of the Pd clusters (Supplementary Figs. 17–19).

**Fig. 4:**
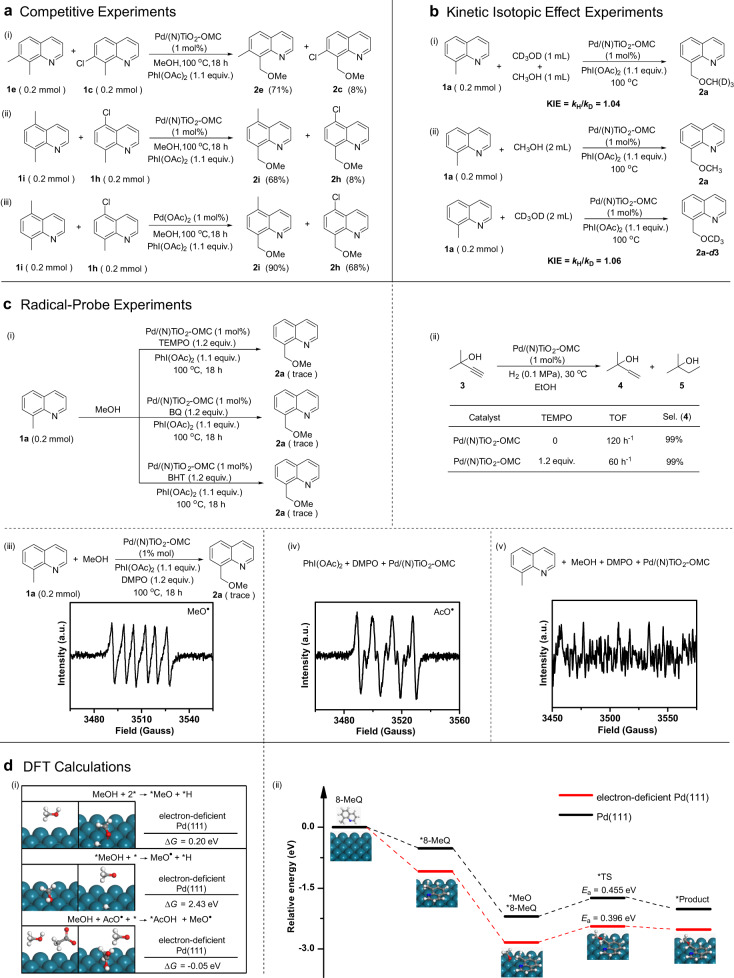
Reaction mechanism. **a** Intermolecular competition experiments between substituted quinolines showing the effects of electron-withdrawing or donating substituents on the Pd catalyzed direct methoxylation of quinolines. **b** Kinetic isotope effects measured using deuterated substrates under (i) intermolecular competition and (ii) independent experiments. **c** Radical trapping experiments and EPR spectra. (i) TEMPO, BQ and BHT were used as radical scavengers to quench the reaction. (ii) Excluding the complete poisoning effect of the radical scavenger of TEMPO on the Pd nanocatalyst in a reaction which is apparently not a radical-involving reaction (hydrogenation of 2-methyl-3-butyn-2-ol). (iii–v) EPR spectra of radical anions when the radical spin trapping reagent DMPO was added to (iii) the reaction system, (iv) in the absence of methanol, and (v) in the absence of PhI(OAc)_2_. **d** DFT calculations. (i) Generation of methoxyl radicals by Pd-catalyzed radical relay reaction over an electron-deficient Pd(111) surface. (ii) Reaction paths and energy landscape of the direct methoxylation of 8-MeQ over Pd(111) and an electron-deficient Pd(111) surface.

As shown in Fig. 3c, an extended range of substituted quinolines has been investigated, and both electron-deficient and electron-rich heteroarenes were well tolerated (yield of 61–86%). An intermolecular competition experiment between electron-rich substrates and electron-deficient substrates was also performed. Electron-rich quinolines (**1e** and **1i**) react preferentially compared to electron-deficient ones (**1c** and **1h**). The molar ratios of products **2e**:**2c** and **2i**:**2h** were 71:8 and 68:8, respectively (Fig. 4a). In contrast, no obvious competition between electron-rich and electron-deficient quinolines was observed in the presence of homogeneous Pd(OAc)_2_. These phenomena indicate the different interactions between the substrate and Pd in heterogeneous and homogenous catalysis. In a kinetic isotope effect (KIE) test, the *k*_H_/*k*_D_ value for direct oxidative coupling was estimated to be approximately 1.0, both in competitive and independent experiments with methanol and methanol-*d*4 (Fig. 4b). As a result, the O–H bond cleavage in methanol may not be involved in the rate-determining step. The product in the presence of methanol-*d*4 is **2a**-*d*3, confirming that the oxidative coupling methoxyl group originates from methanol. Experiments were performed at different initial concentrations of **1a**, and showed a linear increase in *r*_0_ in the range 17~149 mmol mmol_Pd_^−1^ h^−1^, indicating a first-order rate equation for **1a** (Supplementary Fig. 20). The apparent activation energies (*E*_a_) for the catalysts are determined from the Arrhenius plots (Fig. 3d and Supplementary Fig. 21). The low *E*_a_ value for both Pd/(N)TiO_2_-OMC (17.9 kJ mol^−1^) and Pd/TiO_2_-OMC (27.7 kJ mol^−1^) may be related to a radical-involved reaction^58,59^. In comparison, *E*_a_ dramatically increases to 40.2 kJ mol^−1^ for Pd/SBA-15, implying a radical-free Pd^II^-Pd^IV^ mechanism due to the leaching of soluble Pd species, as reported^16,57^.

Radical scavengers including 2,2,6,6-tetramethyl-1-piperidinyloxy (TEMPO), 1,4-benzoquinone (BQ), and 2,6-di-tert-butyl-4-methylphenol (BHT) were then added to the reaction (Fig. 4c, i) and all were found to almost completely quench it. One may argue that these radical scavengers are often used as a poisoning agent for Pd nanocatalysts. A parallel experiment, which is apparently not a radical-involving reaction, was carried out. TEMPO does show a poisoning effect on the Pd nanocatalyst in the hydrogenation of 2-methyl-3-butyn-2-ol, but a moderate activity remains (Fig. 4c, ii). Therefore, the complete loss of activity in the direct oxidative coupling indicates the reaction is expected to be a radical process. EPR spectroscopy experiments were performed to investigate the radical intermediate produced. With the addition of the radical spin trapping reagent 5,5-dimethyl-1-pyrroline-*N*-oxide (DMPO), a mixed signal of DMPO trapping methoxyl radicals (*g* = 2.0053, *a*_N_ = 13.9 G, *a*_H_ = 7.7 G) was identified (Fig. 4c, iii). It can therefore be concluded that methoxyl radicals are generated in the reaction. However, acetoxyl radicals (*g* = 2.0096, *a*_N_ = 13.8 G, *a*_H_ = 10.5 G) can be detected in a methanol-free system, which may be caused by the homolytic cleavage of the weak I−O bond of PhI(OAc)_2_ in the presence of heterogeneous Pd^60,61^, and no free radicals are generated in the absence of PhI(OAc)_2_ (Fig. 4c, iv and v). The release of the methoxyl radicals may involve the decomposition of PhI(OAc)_2_ to acetoxyl radicals and subsequent reaction with the adsorbed methanol on the Pd surface. In addition, when [Ph2I]OTf and [Ph_2_I]BF_4_ are used, they both easily generate aryl radicals in the reaction and produce arylated products due to electrophilic substitution reactions between the aryl radicals and the substrate. No alkoxylation products are produced. Therefore, PhI(OAc)_2_ is specific for the current study. A thorough investigation of the radical initiator is needed in the future.

### DFT calculations

The catalyst surface was first constructed by density functional theory (DFT) calculations. Charge transfer at the interface between the Pd(111) and TiO_2_(101) or N-doped TiO_2_(101) with/without O_v_ was studied (Supplementary Table 4). On a TiO_2_(101) surface, O withdraws an electron from TiO_2_ due to the electronegativity difference. When the surface contains O_v_, Pd prefers locate on the top of the O_v_ and the net charge between Pd and TiO_2_ can be neglected, similar to our XPS result for Pd/TiO_2_-OMC. If the O_v_ concentration is high (in this case a large percentage of oxygen vacancies is not associated with Pd atoms, denoted as Pd/TiO_2_(O_v_)), the charge transfer occurs from TiO_2_ to Pd which is true for a Pd/anatase TiO_2_ catalyst with abundant O_v_ as reported in the literature^9^. Different results have been observed for a N-doped TiO_2_(101) surface. In this case, the net charge is calculated to transfer from Pd to TiO_2_ for N-doped TiO_2_(101) even with abundant O_v_, indicating an electron-deficient surface, in good agreement with the previous XPS and XAS results (Supplementary Table 4). As a result, the role of electron-deficient Pd nanocrystals on the direct methoxylation of C–H bonds is simulated.

DFT calculations show that methanol adsorbs on an electron-deficient Pd(111) surface. However, the calculated energy barrier for the dissociative adsorption of methanol is high, and O–H bond dissociation by the metal alone is unlikely (Fig. 4d, i), as has been reported^62,63^. In addition, the adsorbed methanol cannot release a radical. On the contrary, the presence of an acetoxyl radical can facilitate O–H bond activation by single electron transfer. A diffusible methoxyl radical is relayed, while acetic acid is formed on the surface and desorbed which can be detected by gas chromatography (GC). The energetics of methoxyl radical reactivity with the benzylic C–H bond of 8-MeQ was also evaluated (Fig. 4d, ii). The 8-MeQ molecule prefers a flat geometry on an electron-deficient Pd(111) surface (Supplementary Fig. 22). The activation of the C–H bonds at the benzylic sites of 8-MeQ is indicated by the elongation of the bonds on an electron-deficient Pd surface (1.112 Å), and this elongation is dominant compared with a clean Pd(111) surface (Supplementary Table 5). The simultaneous attacking of C–H bonds at the benzylic sites of 8-MeQ by the methoxyl radical and coupling on the electron-deficient Pd(111) surface is the rate-limiting step, with a free energy change of 0.396 eV. In comparison, a larger free energy change of 0.455 eV for this step is observed for a pure Pd(111) surface.

## Discussion

It has been reported that the mechanism of the homogenous Pd-catalyzed oxidative coupling of C(*sp*^3^)–H bonds with PhI(OAc)_2_ involves the following steps: (i) ligand-directed C–H activation to generate a cyclopalladated intermediate, (ii) two-electron oxidation of the palladacycle to generate a Pd^IV^ species possibly with dimeric Pd^III^–Pd^III^ intermediates, and (iii) C–O bond-forming reductive elimination to release the product (Supplementary Fig. 23)^16–19,64^. With the continuous development of homogenous systems including various Pd catalysts and organic ligands, directing agents or mediators, there are many ways the substrate can be used for the Pd catalyzed alkoxylation of C(*sp*^3^)–H bonds^4,65,66^. Substrates include aromatic compounds such as benzo[*h*]quinoline^8,16–18,57^, 2-phenylpyridine^4,8,16–18,57^, benzoxazole^67^, benzothiazole^67^, pyrazines^65^, quinazolinones^65^, aromatic oximes^4,65,68^, and toluene^69^. Heterogeneous catalysts may behave differently from homogeneous ones because the key issues are surface adsorption and reaction. The adsorption and reaction occurring on the surface of Pd has been proved by hot filtration and SH-SBA-15 solid trapping experiments. Pd may increase the formation of radicals by the activation of an oxidant, such as PhI(OAc)_2_. DFT calculations show that methanol is adsorbed on the electron-deficient Pd surface. A diffusible methoxyl radical is relayed by single-electron transfer between the released acetoxyl radical from PhI(OAc)_2_ and adsorbed methanol. The O–H bond is dissociated and H is subtracted by an acetoxyl group with the formation of acetic acid.

To give insight on the surface adsorption of 8-MeQ, we first discuss the important effect of the change in the *d* electron density of metal particles since the adsorption or activation of reactants is largely affected by the interaction of *d* electrons with the frontier orbitals of the reactants^70,71^. DFT calculations indicate that 8-MeQ adsorbs parallel to the Pd(111) surface with an activation of the C–H bonds in the methyl group as indicated in Fig. 4d and Supplementary Table 5. The adsorption restriction on vibrational and rotational freedom is reflected by the activation entropy (Δ*S*^0*^)^72^. It is easily seen that a low *E*_a_ is related to a high Δ*S*^0*^ value calculated according to transition state theory (Fig. 3d), which is called the compensation effect^72,73^, indicating a significant increase in the steric constraints on the surface interactions between chemisorbed species, as well as in the binding energy of the molecule to the surface^29,44,58^. A simple correlation between the *d* charge of the heterogeneous Pd catalysts and Δ*S*^0*^ and the TOF value has been shown (Fig. 3d). The bound state for the adsorbed quinolines, which is energetically most favorable for the catalytic reaction, is increased on Pd/(N)TiO_2_-OMC with its *d* electron-deficient Pd, as expected. An intermolecular competition experiment has shown that this reaction is highly dependent on electronic factors. The preferential direct oxidative coupling for electron-rich quinolines compared to electron-deficient ones confirms the preferential adsorption of electron-rich quinolines on an electron-deficient Pd surface. The above mentioned bisarylation method for five-membered heteroarenes that engages heterogeneous Pd nanoparticles as catalysts shows the use of N-, O-, and S-heteroarene as substrates^29^, and the activation of the adjacent C–H bonds. Therefore, other N-, S, and O-containing aromatic compounds such as benzo[*h*]quinoline, 2-phenylpyridine, benzoxazole, and benzothiazole which can be strongly adsorbed on the Pd surface are also possibly available to this system; but the substrate scope is still limited compared to the homogenous catalyst systems because surface adsorption limits the use of a large variety of ligands, directing agents or mediators.

A possible mechanism for the electron-deficient Pd-catalyzed direct alkoxylation of C(*sp*^3^)–H is proposed here in combination with the above results (Fig. 4d and Supplementary Fig. 24). First, 8-MeQ adsorbs on the Pd surface to form *8-MeQ with an activation of the C(*sp*^3^)–H bonds. At the same time, the adsorption of methanol on the electron-deficient Pd surface is facilitated, and the acetoxyl radical produced by the decomposition of PhI(OAc)_2_ is transformed to form a methoxyl radical which then reacts with the adjacent adsorbed *8-MeQ on the Pd surface with radical addition to the C(*sp*^3^)–H and subtraction of a hydrogen atom. Finally, the adsorbed oxidative coupling product is desorbed from the Pd surface to regenerate available active Pd sites. Preliminary DFT calculations support this surface adsorption-induced radical relay mechanism (Fig. 4d).

In summary, we postulate that a heterogeneous Pd-catalyzed oxygen-centered radical relay strategy can provide the basis for the selective cross-coupling of C(*sp*^3^)–H bonds and alcohols using heterocyclic substrates, and create opportunities for the efficient synthesis of chemical structures with no Pd contamination. Pd nanoparticles are supported on a novel ordered mesoporous composite composed of N-doped anatase nanocrystals and ordered mesoporous carbon. The N-doped anatase nanocrystals provide strong interaction with the metal nanocatalyst to generate *d* electron-deficient Pd. Heterogeneous electron-deficient Pd plays three essential roles: (i) PhI(OAc)_2_ decomposes to an acetoxyl radical in the presence of Pd nanoparticles, (ii) the adsorption of methanol on the Pd surface is facilitated in the presence of acetoxyl radicals which are transformed to methoxyl radicals by a radical relay reaction; and (iii) 8-MeQ adsorbs on the electron-deficient Pd, and the methoxyl radicals react with adjacent adsorbed molecules of 8-MeQ on the surface with hydrogen-atom abstraction to form C–O bonds. The reactions proceed efficiently under mild conditions, and show a good activity (TOF of 84 h^−1^) and high selectivity toward ethers (>99%). Negligible Pd leaching and activity loss is observed after 7 catalytic runs. The Pd surface adsorption-induced radical relay method for the direct oxidative coupling of C(*sp*^3^)–H bonds described here represents a valuable demonstration of C–O bond formation heterogeneous catalysis, different from the traditional homogenous cyclopalladated intermediate using Pd^II^/Pd^IV^ cycles. The ability to use stable electron-deficient Pd nanoparticles and the use of C(*sp*^3^)–H heteroarene substrates as the reagent provide key foundations for the pursuit of other chemo-, regio-, and stereoselective C–H functionalization reactions and reducing the risk of metal contamination.

## Methods

### Synthesis of Pd/(N)TiO_2_-OMC

The ordered mesoporous (N)TiO_2_-OMC supports were synthesized by a surfactant-templating method. A typical synthesis is as follows. A Stöber solution was obtained by dissolving 1.1 mL of TiCl_4_ in 1.0 g of distilled water and 10.0 g of ethanol at 0 °C. Then, a clear solution containing 1.5 g of Pluronic F127 (poly(propylene oxide)-*block*-poly(ethylene oxide)-*block*-poly(propylene oxide), PEO_106_PPO_70_PEO_106_, *M*_w_ = 12,600), 1.0 g of deionized water and 8.0 g of ethanol was mixed with the Stöber solution at 40 °C. After stirring the mixture for 30 min, a solution containing 5.0 g of 20 wt% low-polymerized phenolic resins and 0.34 g of urea was added and the mixture was stirred for a further 1 h to produce a transparent claret-colored mixture. The mixture was poured into multiple petri dishes that were immediately placed in an oven at 40 °C. After 24 h, the dishes were heated at 100 °C for further thermopolymerization of the carbonaceous resins. The resulting transparent thin films were then heated at 350 °C for 5 h to remove the template. Further crystallization of titania was accomplished at 600 °C for 1 h under a nitrogen atmosphere to obtain (N)TiO_2_/OMC. The supported palladium catalysts were prepared by a wet impregnation method. In a typical procedure, 0.68 g of an aqueous solution of PdCl_2_ (1.1 wt%) was mixed with 0.4 g of (N)TiO_2_/OMC carrier for 6 h under stirring. The mixture was evaporated, washed, and dried at 50 °C under vacuum. The as-made catalyst was then reduced at 300 °C in 10 *v*% H_2_ in nitrogen for 3 h, and is referred to as the fresh supported catalyst (Pd/(N)TiO_2_-OMC). The Pd concentration was determined by ICP-AES. The synthesis of 0.50 wt% Pd/(N)TiO_2_-OMC, 1.49 wt% Pd/(N)TiO_2_-OMC and 1.99 wt% Pd/(N)TiO_2_-OMC was the same as for Pd/(N)TiO_2_-OMC, except that the amounts of the aqueous solution of PdCl_2_ added were changed to 0.34, 1.02 and 1.36 g, respectively.

For comparison, N-free mesoporous TiO_2_-OMC material and mesoporous silica (SBA-15) were also prepared (Supplementary Information) and these carriers were used to prepare supported Pd catalysts by the above-wet impregnation method and are named Pd/TiO_2_-OMC and Pd/SBA-15.

### Theoretical calculations

The spin-polarized first-principles calculations based on density functional theory (DFT) were performed using the Vienna Ab initio Simulation Package (VASP). The adsorption energy (*E*_ads_) was calculated as: *E*_ads_ = *E*_total_ – *E*_molecule_ – *E*_surface_, where *E*_total_ is the adsorption energy of the molecule on the surface of Pd. The free energies were calculated using the equation: ∆*G* = ∆*E*_DFT_ + ∆*E*_ZPE_ – T∆*S*, where ∆*E*_DFT_ is the DFT energy difference which is equal to “–*E*_ads_” for the dissociation. ∆*E*_ZPE_ and T∆*S* are respectively the zero-point energy correction and the change of entropy, which were obtained from vibration calculations. Details of the DFT calculations are given in the Supplementary Information.

### Characterization

The XRD patterns were obtained using a Rigaku Dmax-3C diffractometer using Cu *K*α radiation (40 kV, 20 mA, *λ* = 0.15408 nm), The TiO_2_ particle sizes were calculated using the Scherrer formula, *D*_TiO2_ = 0.89*λ*/*β*cos*θ*, applied to the (101) diffraction peak in the wide-angle XRD patterns. Structural investigations of the catalysts were performed on a JEM 2100 microscope operated at 200 kV and an FEI Tecnai G2 F20 microscope equipped with a HAADF-STEM detector in TEM and STEM modes. Elemental analyses of the spots, lines, and areas selected in the HAADF-STEM images were performed using a Philips EDX instrument. The EDX spectra were obtained in the STEM mode with a focused electron beam in the sub-nanometer size range. N_2_ physisorption isotherms were recorded at 77 K with a Micromeritics TriStar II 3020 analyser using the Brunauer-Emmett-Teller method to estimate the specific surface areas (*S*_BET_) and the Barrett-Joyner-Halenda (BJH) model to derive the pore volumes (*V*p) and pore size distributions (*D*p). The Pd loadings of the solid catalysts and the leaching of Pd in each catalytic run were determined by a Varian VISTA-MPX ICP-AES instrument. Thermal analysis measurements were performed on a Mettler-Toledo TG/SDTA 851e apparatus. Samples were heated from 30 °C to 900 °C at a rate of 10 °C min^−1^ with a nitrogen flow rate of 50 mL min^−1^. The exposed surface of the supported Pd catalyst was measured by pulsed CO adsorption on a Micromeritics Auto Chem II 2920 system. The catalysts were pretreated in 10 *v*% H_2_/Ar at 100 °C for 1 h then cooled to room temperature in pure He flowing at a rate of 20 mL min^−1^. Experiments were taken by pulsing research grade CO on the pretreated catalyst. Ten pulses were recorded per analysis and referenced to a blank run obtained without adding any solid catalyst. The pulse loop volume used was 0.5 mL with the pulse decay was set to 15 min. A 1:1 CO to metal stoichiometric factor was used for the dispersion calculations. The C, H, N, and O contents were measured using a Vario EL III elemental analyzer. The XPS measurements were recorded on a Perkin-Elmer PHI 5000 CESCA system. The samples were evacuated in a load-lock chamber and then transferred to the analysis chamber (10^−9^ mbar). The C 1 *s* peak was used as an internal standard for the determination of the peak positions and was corrected to 284.6 eV. The Pd binding energies were fitted by peak fitting techniques. The X-ray absorption fine structure spectra data (Pd *L*_3_- and *K*-edge) were collected at 4B7A station in the Beijing Synchrotron Radiation Facility (BSRF) and BL14W1 station in the Shanghai Synchrotron Radiation Facility (SSRF), respectively. The data processing was performed using the ATHENA program. The Pd EXAFS data of the sample were fitted by a fast Fourier inverse transformation (IFEFFIT) in *R* space using a software package. The difference in the number of 4*d* charges between the samples and Pd/SBA-15 was evaluated from the Pd *L*_3_-edge XANES. Detailed information is provided in Supplementary methods. EPR spectra of the materials were taken on a Bruker E580 instrument at room temperature. The frequency was 9.84 GHz and the microwave power was 2.0 mW.

### Catalysis tests

The oxidation reactions were carried out in round-bottom flasks. In a typical reaction, 0.22 mmol of iodosobenzene diacetate and 21 mg of powdered catalyst were added to a mixture of 0.2 mmol of 8-MeQ and 2 mL of methanol. The suspension was at atmospheric pressure, the stirring speed was fixed at 800 rpm and the temperature was kept at 100 °C. After the reaction, both the solid catalyst and mother liquor were extracted with ethyl acetate. The organic phases were combined and analyzed to determine the conversion and yield by gas chromatography-mass spectrometry (GC-MS, 7890B-5977) or GC (Agilent 7890B) using a DB-Wax column. The solid catalyst was recovered for reuse by washing with water and drying at 80 °C overnight under a vacuum. The analysis was repeated at least three times with ±5% experimental errors (standard deviation). The carbon balance for all tests was above 95%. The identification of the product was also conducted by ^1^H nuclear magnetic resonance spectra (^1^H NMR) measurements on a Bruker DRX 400 spectrometer using tetramethylsilane as the internal standard.

Preliminary experiments were performed by changing stirring speeds from 600 to 1000 rpm to study the diffusion limitations. The effect of mass transfer on the reaction rate was studied by changing the surface concentrations of the metal but with similar dispersions, known as the Madon–Boudart (MB) test. Three additional samples with different total metal contents (0.5–2 wt%) were synthesized.

Solid mercapto-functionalized ordered mesoporous silica was used to trap the soluble metal species that had leached into solution. Typically, 49 mg of SH-SBA-15 was added to the reaction flask prior to the addition of the reaction solution to give a molar ratio of S:Pd = 35:1. Hot filtration was also used at different time intervals to determine the 8-MeQ conversion and metal leaching.

The recycling tests were carried out as follows. Each time the catalyst was recycled, the oxidation reaction was performed under the same conditions. To ensure the same amount of catalyst was present during each cycle, multiple parallel tests were performed. The solutions after each run were collected and combined to determine the metal leaching.

To investigate the radical reaction mechanism, 1.2 equivalent of TEMPO (or BQ, or BHT) was added to the reaction batch which was used as a radical scavenger. The capture of active radicals generated during the reaction was examined by EPR spectra (Bruker ELEXSYS 500 spectrometer) using DMPO as a spin trapping agent (Supplementary methods).

A compared reaction of hydrogenation of 2-methyl-3-butyn-2-ol was carried out. 0.2 mmol of 2-methyl-3-butyn-2-ol, 2 mL of ethanol, and 1% mol of Pd/(N)TiO_2_-OMC were added to a round bottom flask at 30 °C and sealed by an atmospheric balloon full of 100% H_2_. The conversion and yield were analyzed and determined by GC (Agilent 7890B) using a HP-INNOWax capillary column.

A competition reaction of the deuterium KIE study was used by mixing 1 mL of CH_3_OH and 1 mL of CD_3_OD in the reaction batch. After stirring for 30 mins, the catalyst was quickly removed, and the product was purified by column chromatography on silica gel. The yield was determined by ^1^H NMR.

## Supplementary information

Supplementary Information

## Data Availability

The data that support the findings of this study are available from the corresponding authors upon reasonable request.
