# Supplementary material for: Palladium catalyzed radical relay for the oxidative cross-coupling of quinolines
Source: Nat Commun. 2022 Jul 19;13:4180. doi: 10.1038/s41467-022-31967-0 (PMC9296488; doi:10.1038/s41467-022-31967-0)
Supplement: Supplementary file 1 — Supplementary Information [file 41467_2022_31967_MOESM1_ESM.pdf]

Supplementary Information

Palladium catalyzed radical relay for the oxidative cross-coupling of quinolines

Xiaorui Zhao[1,2#], Xiaojuan Zhu[1#], Kang Wang[1], Junqian Lv[1], Shangjun Chen[1], Guohua Yao[1], Junyu Lang[3], Fei Lv[1], Yinghui Pu[4], Ruoou Yang[5], Bingsen Zhang[4]*, Zheng Jiang[6]*, Ying Wan[1]*

[1]The Education Ministry Key Laboratory of Resource Chemistry, Joint International Research Laboratory of Resource Chemistry of Ministry of Education, Shanghai Key Laboratory of Rare Earth Functional Materials, and Shanghai Frontiers Science Center of Biomimetic Catalysis, Shanghai Normal University, Shanghai, P. R. China.

[2]School of Chemistry and Chemical Engineering, Taishan University, Shandong, P. R. China.

[3]School of Physical Science and Technology, Shanghai Tech University, Shanghai, P. R. China.

[4]Shenyang National Laboratory for Materials Science, Institute of Metal Research, Chinese Academy of Sciences, Shenyang, P. R. China.

[5]State Key Laboratory of Materials Processing and Die & Mould Technology, School of Materials Science and Engineering, Huazhong University of Science and Technology, Hubei, P. R. China.

[6]Shanghai Synchrotron Radiation Facility, Zhangjiang National Lab, Shanghai Advanced Research Institute, Chinese Academy of Sciences, Shanghai, P. R. China.

[#]These authors were equal major contributors: Xiaorui Zhao and Xiaojuan Zhu.

*To whom correspondence should be addressed. E-mail: bszhang@imr.ac.cn; jiangzheng@sinap.ac.cn; ywan@shnu.edu.cn.

Contents

Supplementary Tables 3
Supplementary Figures 8
Supplementary Methods 32
^{1}H NMR Data 36
Supplementary References 43

Supplementary Table 1. The structural and textural properties of Pd nanocatalysts. For comparison, the properties for Pd/SBA-15 and Pd/C and the metal-free ordered mesoporous anatase-carbon composite carrier are also provided.

**Sample**	**Nitrogen content[a] (*wt*%)**	**TiO_2 content[b] (*wt*%)**	**Pd loading[c] (*wt*%)**	**τ^d (%)**	**d_{Pd}^d (nm)**	**d_{Pd}^e (nm)**	**S_{BET} ($m^2\ g^{-1}$)**	**V_p ($cm^3\ g^{-1}$)**	**D_p (nm)**
Pd/(N)TiO_2-OMC	1.23	58	0.99	51	2.2	2.3	402	0.29	4.3
Pd/(N)TiO_2-OMC-R[f]	n.p.[g]	n.p.	0.97	45	2.4	2.4	376	0.24	4.1
0.50-Pd/(N)TiO_2-OMC	1.23	58	0.50	51	2.2	n.p.	421	0.31	4.3
1.49-Pd/(N)TiO_2-OMC	1.23	58	1.49	51	2.2	n.p.	398	0.28	4.3
1.99-Pd/(N)TiO_2-OMC	1.23	58	1.99	51	2.2	n.p.	387	0.26	4.3
Pd/TiO_2-OMC	0	60	0.99	47	2.3	2.5	395	0.27	4.4
Pd/SBA-15	0	0	1.00	37	2.9	3.1	830	1.13	9.0
Pd/C	0	0	5.03	20	5.5	3.9	1333	0.82	1.4
(N)TiO_2-OMC	1.27	59	0	0	0	0	476	0.33	4.4
TiO_2-OMC	0	60	0	0	0	0	460	0.32	4.5

[a]Measured by element analysis;
[b]Measured by TG;
[c]Measured by ICP-AES;
[d]Obtained from CO chemisorption, d_{Pd} is the average diameter of Pd nanoparticles;
[e]Estimated from the TEM images with the size histograms counted from at least 200 nanoparticles;
[f]The Pd/(N)TiO_2-OMC catalyst after the seventh catalytic run;
[g]Not provided.

Supplementary Table 2. *K*-edge k^2-weighted EXAFS data fittings of Pd samples.

**Sample**	**Shell**	**CN**[a]	***R*** **(Å)**[b]	$\Delta\sigma^2$***10**^{3} **(Å**^{2}**)**[c]	***R*-factor (%)**[d]
Pd/(N)TiO_2-OMC	Pd-Pd	1.8±1.0	2.88±0.04	13.4±5.9	1.9
	Pd-Ti	0.4±0.3	2.64±0.03	0.1±0.6	
	Pd-O/N	3.8±0.3	2.14±0.03	9.0±0.3	
Pd/TiO_2-OMC	Pd-Pd	6.5±0.9	2.75±0.01	10.6±1.6	1.7
	Pd-Ti	1.8±0.7	2.68±0.02	9.0±4.6	
	Pd-O	0.8±0.3	2.00±0.03	8.7±4.4	
Pd foil	Pd-Pd	12	2.74	/	/
PdO	Pd-O	4	2.01	/	/
	Pd-Pd	4	3.04	/	/

[a]CN is the coordination number;
[b]*R* is the distance between absorber and backscattered atoms;
[c]$\Delta\sigma^2$*10^3 is the Debye-Waller factor to account for both thermal and structural disorders;
[d]*R*-factor indicates the goodness of the fit.

Supplementary Table 3. Leaching of Pd in each catalytic run.

Runs	Pd/SBA-15	$Pd/(N)TiO_2$-OMC
	Concentration of Pd in filtrate (ppm)[a]	Concentration of Pd in filtrate (ppm)
1	19	BDL[b]
2	22	BDL
3	/	BDL
4	/	BDL
5	/	BDL
6	/	BDL
7	/	BDL

[a]The concentration of Pd in the filtrate after each run was determined by ICP-AES.

[b]Below the detection limit.

Reaction conditions: 1 mol% Pd catalyst; 0.2 mmol of 8-MeQ; 1.1 equivalent of iodobenzene diacetate; 2 mL of methanol; 100 ^{o}C; 800 rpm; atmospheric pressure; in air.

Supplementary Table 4. Calculated charge transfer at the interface between the Pd(111) and TiO_2(101) or N-doped TiO_2(101).

Model	Side view	Bader net charge of Pd (e)	Net electric charge density (10^{-2} e $atom_{Pd}^{-1}$)
Pd/TiO_2	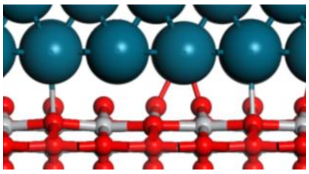	-0.523	-0.73
Pd/TiO_2(O_v)	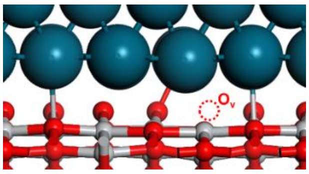	0.214	0.30
Pd/TiO_2(O_v-Pd)	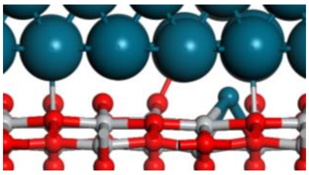	-0.025	-0.03
Pd/(N)TiO_2	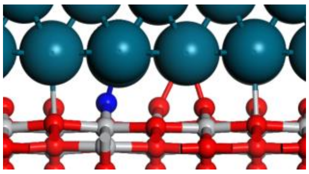	-0.793	-1.10
Pd/(N)TiO_2(O_v)	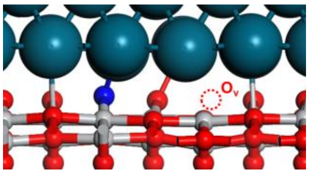	-0.029	-0.04
Pd/(N)TiO_2(O_v-Pd)	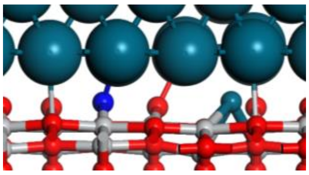	-0.270	-0.38

The charge transfer at the interface between the Pd(111) and TiO_2(101) or N doped TiO_2(101) was studied by the Bader analysis that helps to estimate the total charge on atoms[1]. Based on our experiment results, N-doping, an O vacancy (O_v) or/and an interstitial Pd atom might be present at the interface. For simplification, six models were studied. First, a model of Pd/TiO_2 was built by a super cell resampling method. Subsequently, one O atom was removed from TiO_2 to construct the model of Pd/TiO_2(O_v), an O atom was replaced by a N atom to construct the model of Pd/(N)TiO_2, an O atom was removed in (N)TiO_2 to construct the model of Pd/(N)TiO_2(O_v). An interstitial Pd atom at the O_v site was introduced in Pd/TiO_2(O_v) and Pd/(N)TiO_2(O_v), and are denoted Pd/TiO_2(O_v-Pd) and Pd/(N)TiO_2(O_v-Pd), respectively. All these structures were fully optimized and are shown in this Table. The net electric charge density was calculated using the equation:

$$\text{Net electric charge density} = \frac{\text{Bader net charge of Pd}}{\text{Number of Pd atoms}} \quad (1)$$

To simplify and highlight the effect of an electron-deficient Pd surface, the net electric charge density was set to -1.10×10^{-2} e $atom_{Pd}^{-1}$ for Pd/(N)TiO_2 in subsequent calculations.

Supplementary Table 5. The calculated bond lengths and adsorption energies of 8-methylquinoline (8-MeQ) adsorbed on different surfaces.

**Surface**	**$d_{C7–C8}$ (Å)**[a]	**$d_{C8–H1}$ (Å)**[b]	**$d_{C8–H2(3)}$ (Å)**[c]	**E_{ads} (eV)**
Pd(111)	1.503	1.106	1.098 (1.097)	-0.522
electron-deficient Pd(111)	1.498	1.112	1.099 (1.097)	-1.090

[a]The length of the C_7–C_8 bond in adsorbed 8-MeQ;

[b]The length of the C_8–H_1 bond of the methyl group in adsorbed 8-MeQ. H_1 is the H atom close to the Pd surface in the adsorbed 8-MeQ molecule;

[c]The lengths of the C_8–H_2 or C_8–H_3 bonds of the methyl group in adsorbed 8-MeQ. H_2 and H_3 are the atoms furthermost from the Pd surface in the adsorbed 8-MeQ molecule.

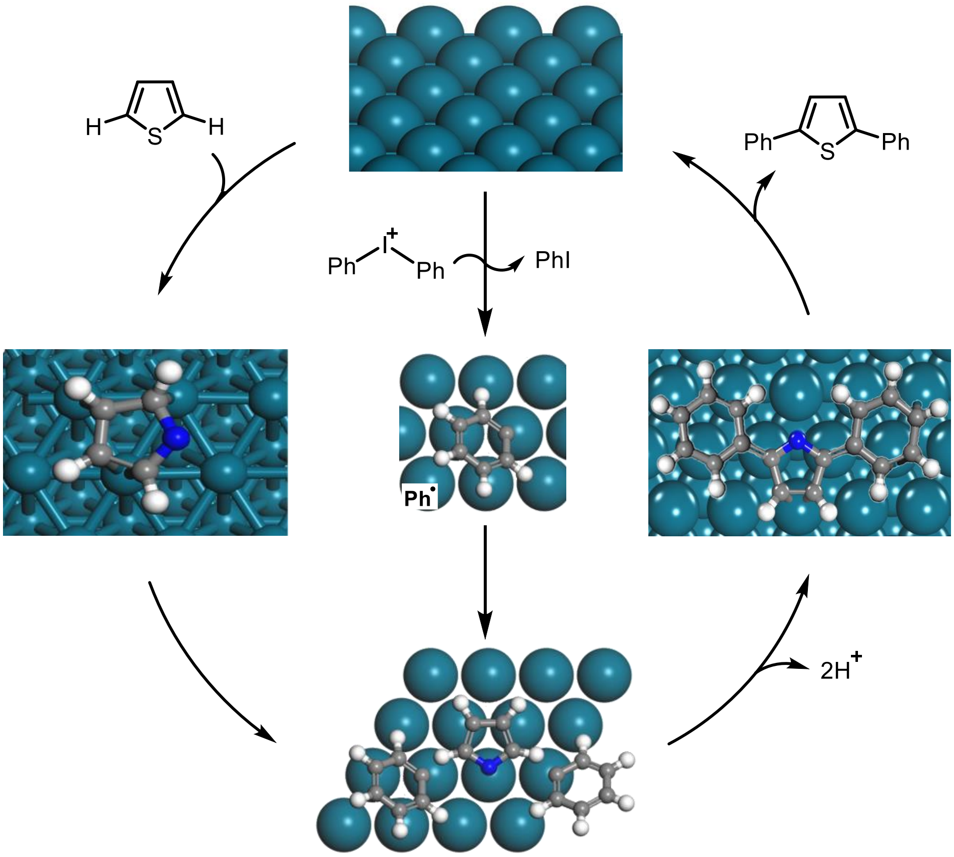

Supplementary Fig. 1. Possible mechanism for the bisarylation transformation. Possible mechanism for the direct bisarylation over a heterogeneous C,N-modified Pd nanocatalyst supported on ordered mesoporous carbon (C,N-Pd/OMC) with free thiophene as an example. The parallel adsorption of thiophene over Pd clusters induces the simultaneous activation of the target C2(sp^2)–H and C5(sp^2)–H in thiophene, and subsequent bisarylation by aryl radicals on the surface. This strategy is distinct from the homogenous ones, including $Pd^{0/II}$ catalysis which may involve the electrophilic attack of Pd on the arene, transmetallation and reductive elimination[2,3]; and $Pd^{II/IV}$ catalysis which may involve the formation of a substrate-Pd^{II} complex through electrophilic palladation, and subsequent oxidative arylation[4]. The single-site electron transfer process yields the monoarylated product.

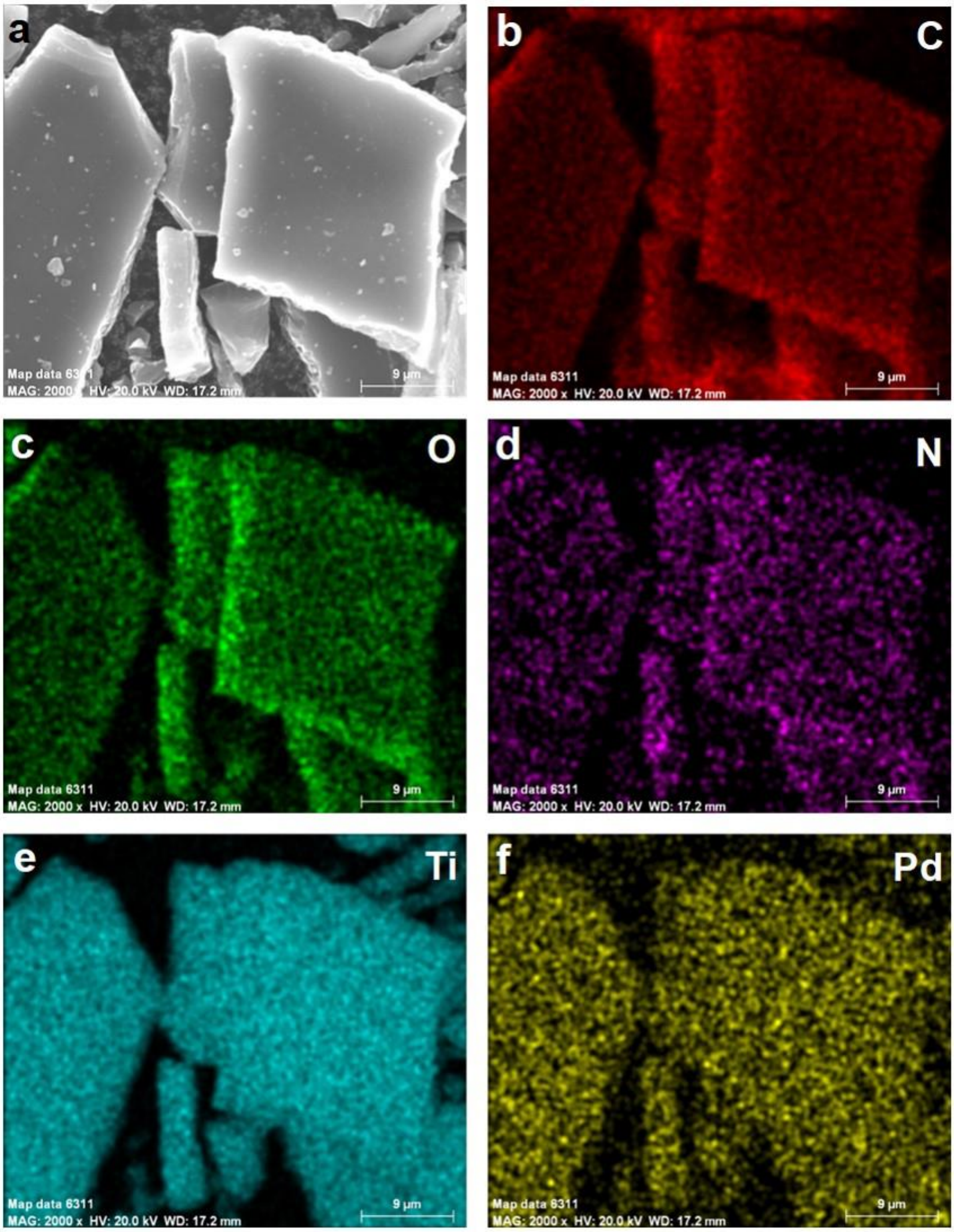

Supplementary Fig. 2. Compostions. (**a**) Representative scanning electron microscopy (SEM) image for Pd/(N)TiO_2-OMC and (**b-f**) the corresponding C, O, N, Ti and Pd EDS elemental mapping results.

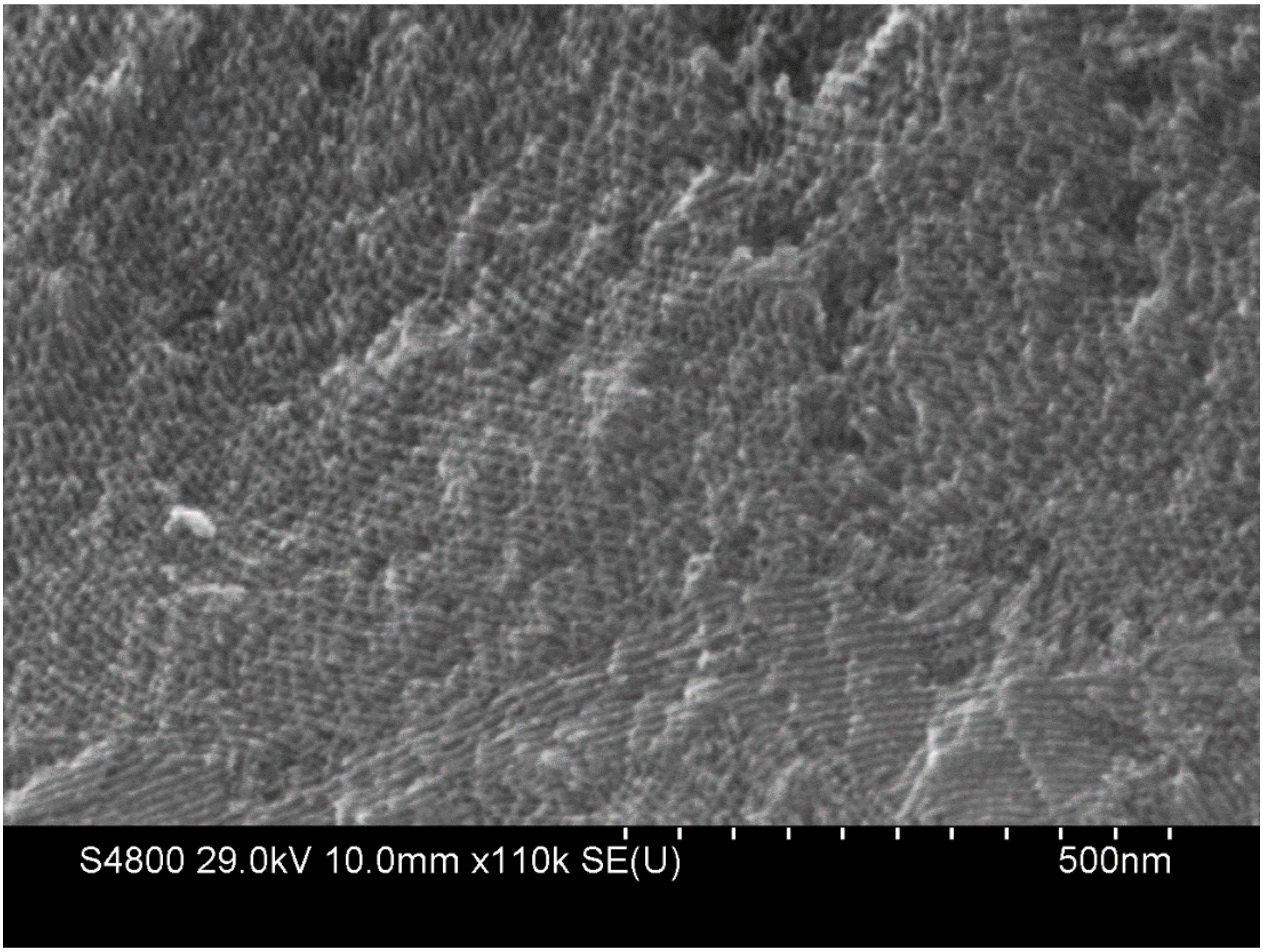

Supplementary Fig. 3. Opened ordered mesopores. Representative high-resolution SEM (HRSEM) image for Pd/(N)TiO_2-OMC.

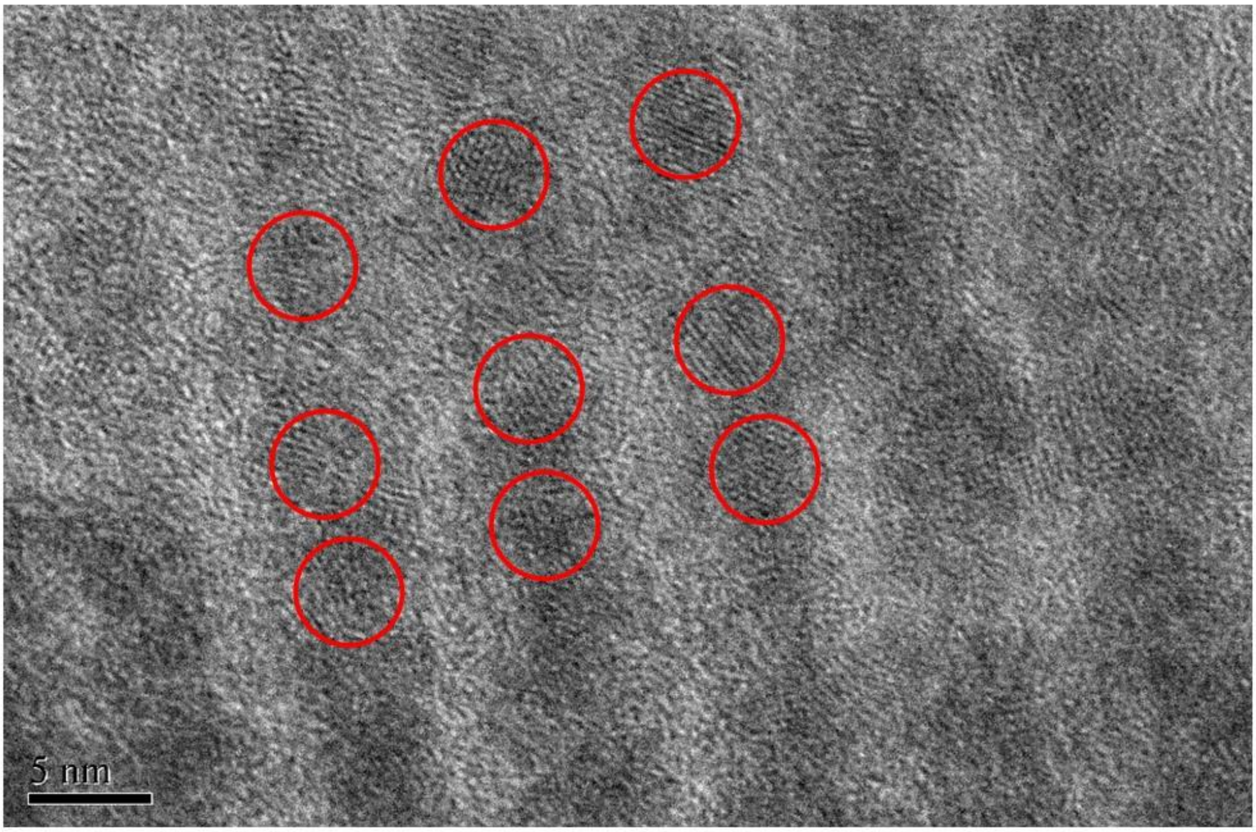

Supplementary Fig. 4. Pore wall compostion. Representative high-resolution transmission electron microscope (HRTEM) image for the (N)TiO_2-OMC. Red circles show TiO_2 nanoparticles.

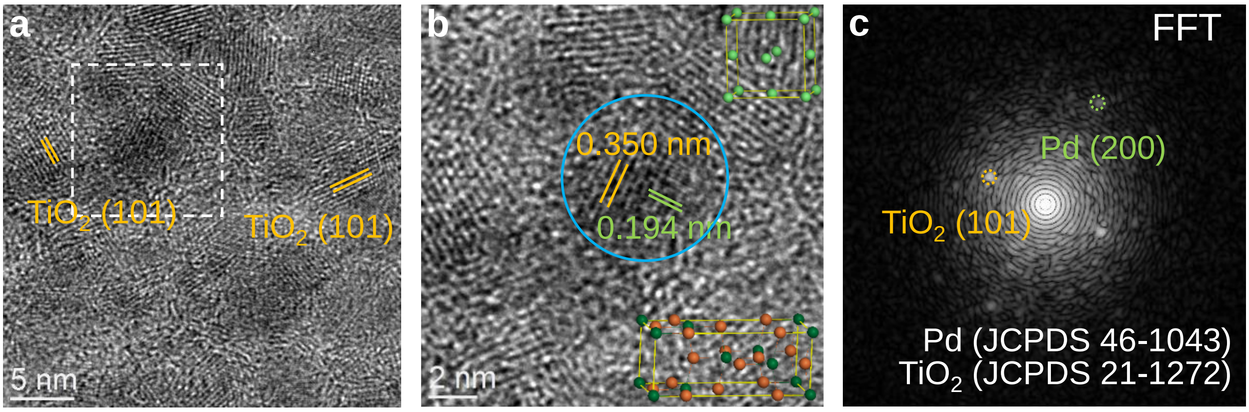

Supplementary Fig. 5. Structure of Pd/(N)TiO_2-OMC. (**a**) HRTEM image of Pd/(N)TiO_2-OMC. (**b**) Enlarged view of the white square in **a**. The top-right and bottom-right insets correspond to Pd and TiO_2 unit cells, respectively. (**c**) Selected-area FFT patterns of Pd and TiO_2 from the area in the blue circle in **b**.

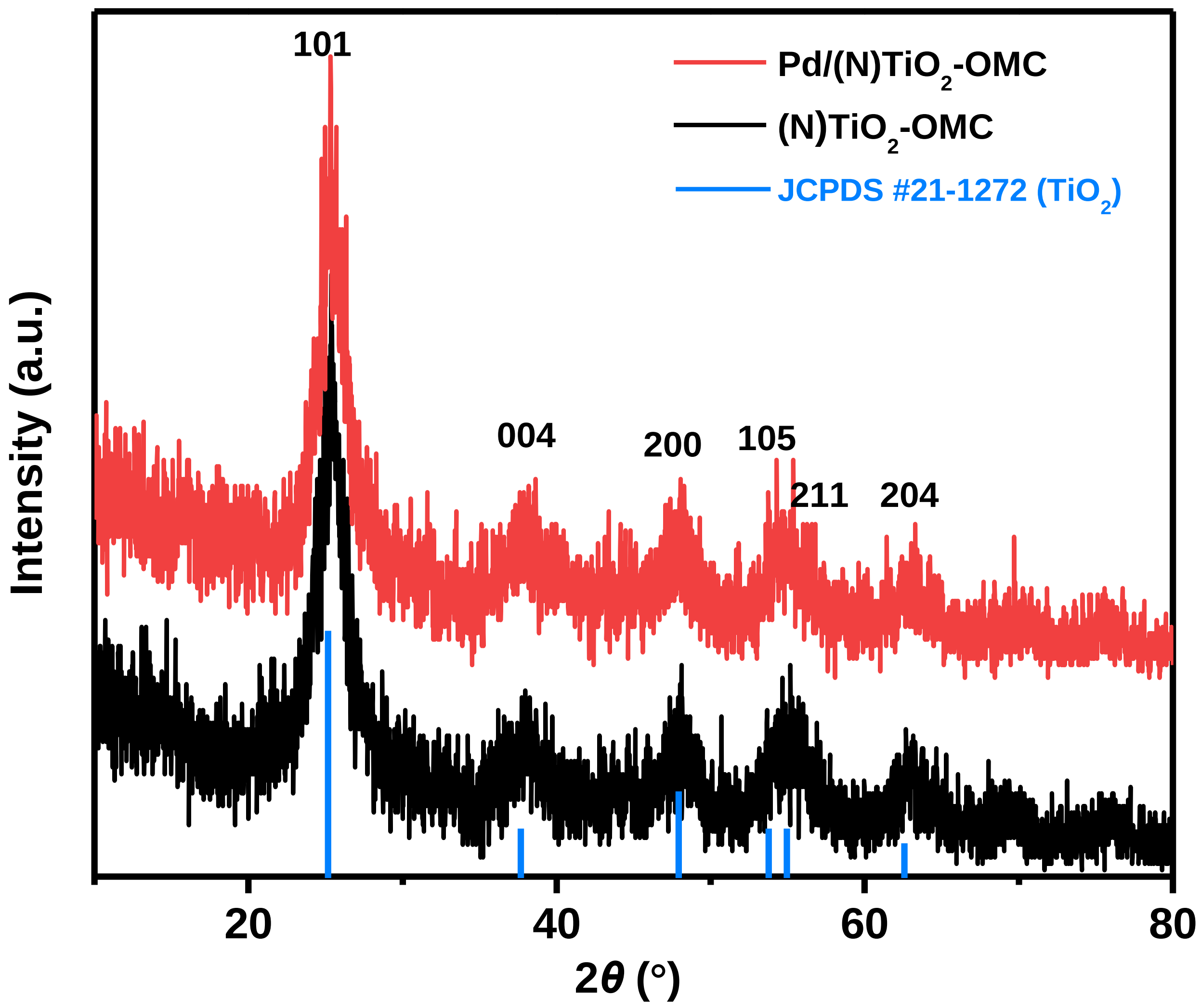

Supplementary Fig. 6. Crystal Structure. Wide-angle X-ray diffraction (XRD) patterns of Pd/(N)TiO_2-OMC and (N)TiO_2-OMC.

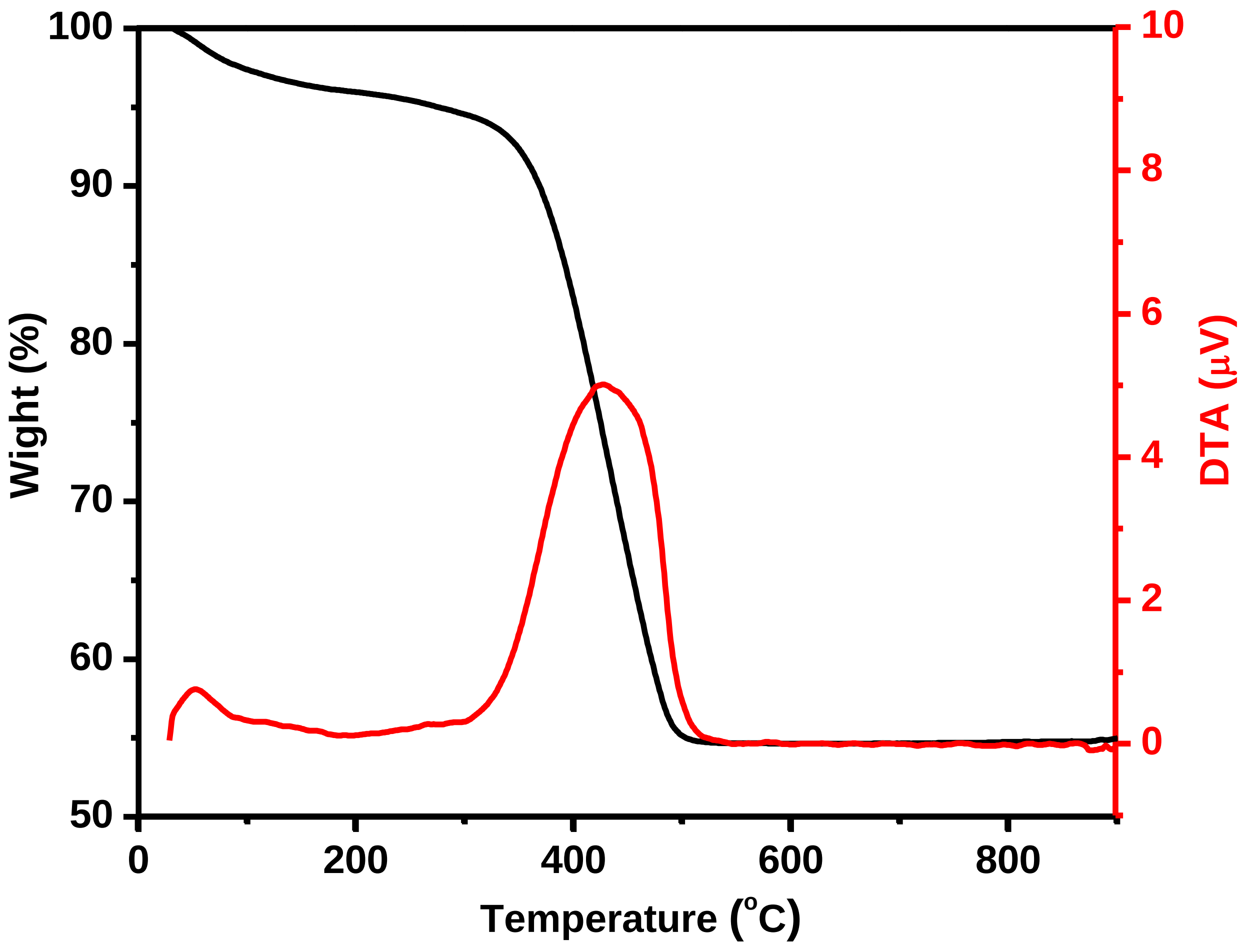

Supplementary Fig. 7. Thermal analysis. Thermogravimetric and differential thermal analysis (TG/DTA) carried out in a nitrogen atmosphere for as-made (N)TiO_2-OMC.

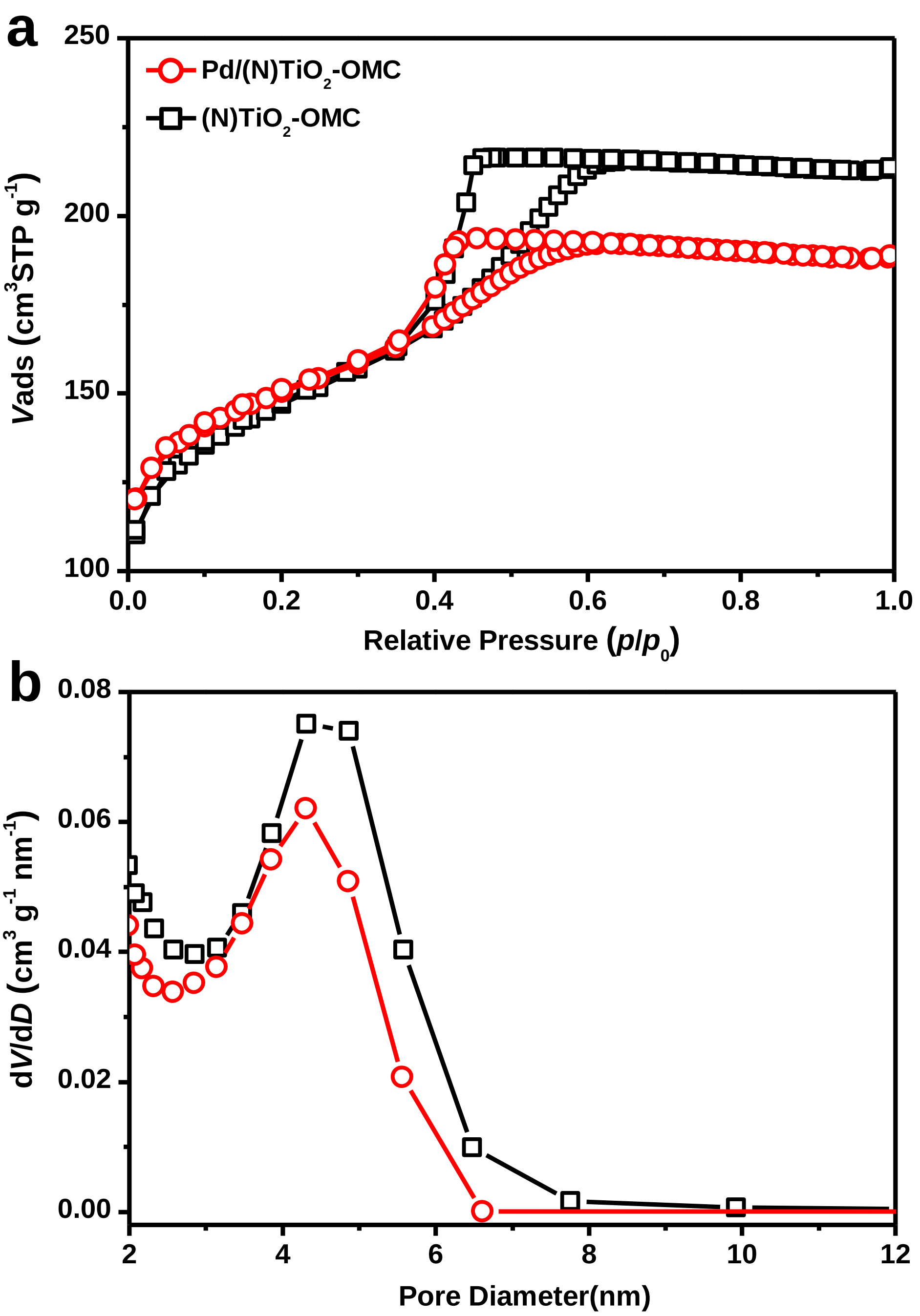

Supplementary Fig. 8. Pore structure for the nanocatalyst and carrier. (**a**) N_2 sorption isotherms and (**b**) pore size distribution curves for Pd/(N)TiO_2-OMC and (N)TiO_2-OMC.

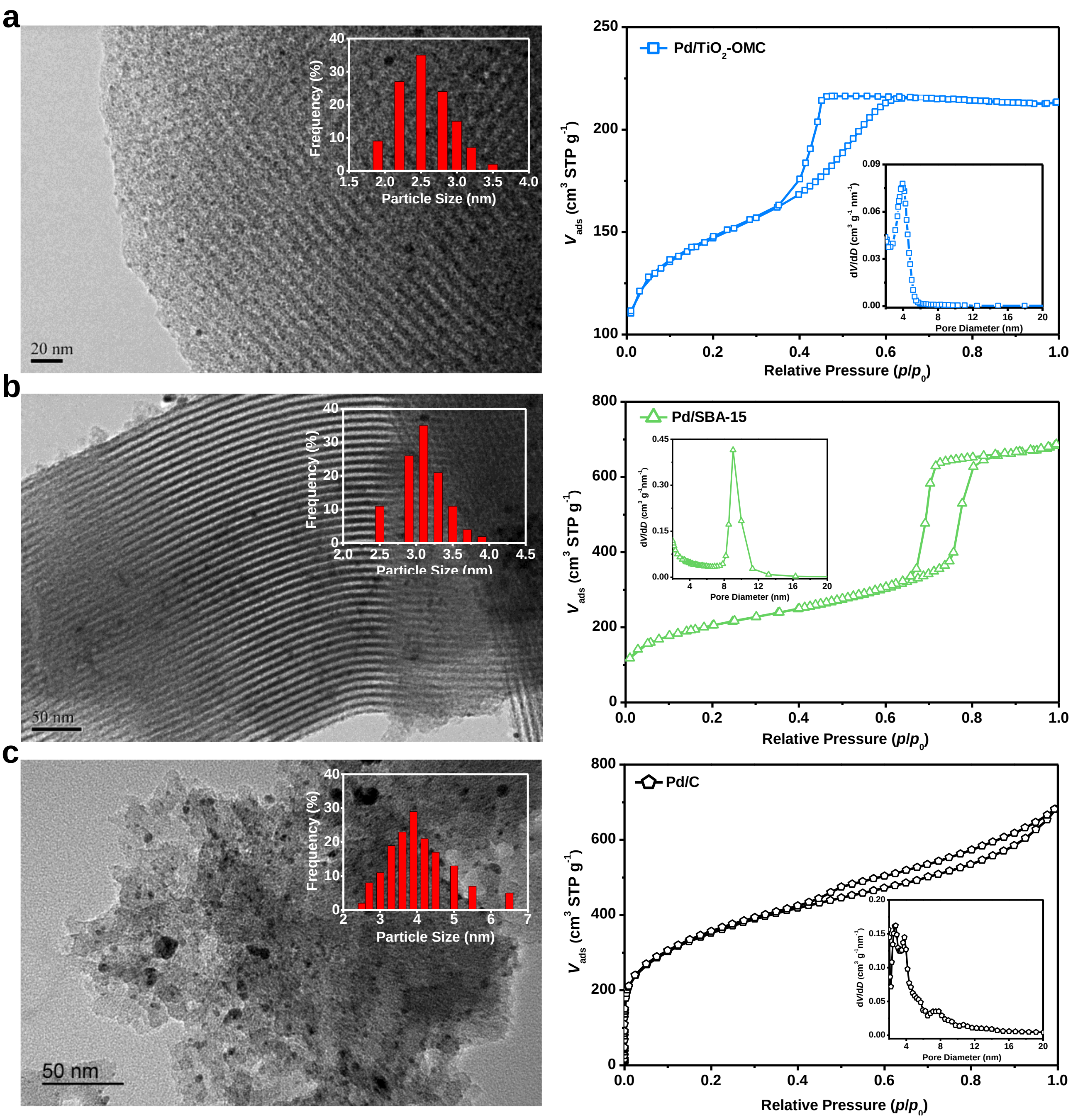

Supplementary Fig. 9. Structure of reference solid nanocatalysts. TEM images and N_2 sorption isotherms for Pd/TiO_2-OMC (**a**), Pd/SBA-15 (**b**) and Pd/C (**c**). The insets in the TEM images are particle size distributions of the corresponding Pd catalysts, counting at least 200 nanoparticles. The insets in N_2 sorption isotherms are the pore size distribution curves for Pd/TiO_2-OMC, Pd/SBA-15 and Pd/C.

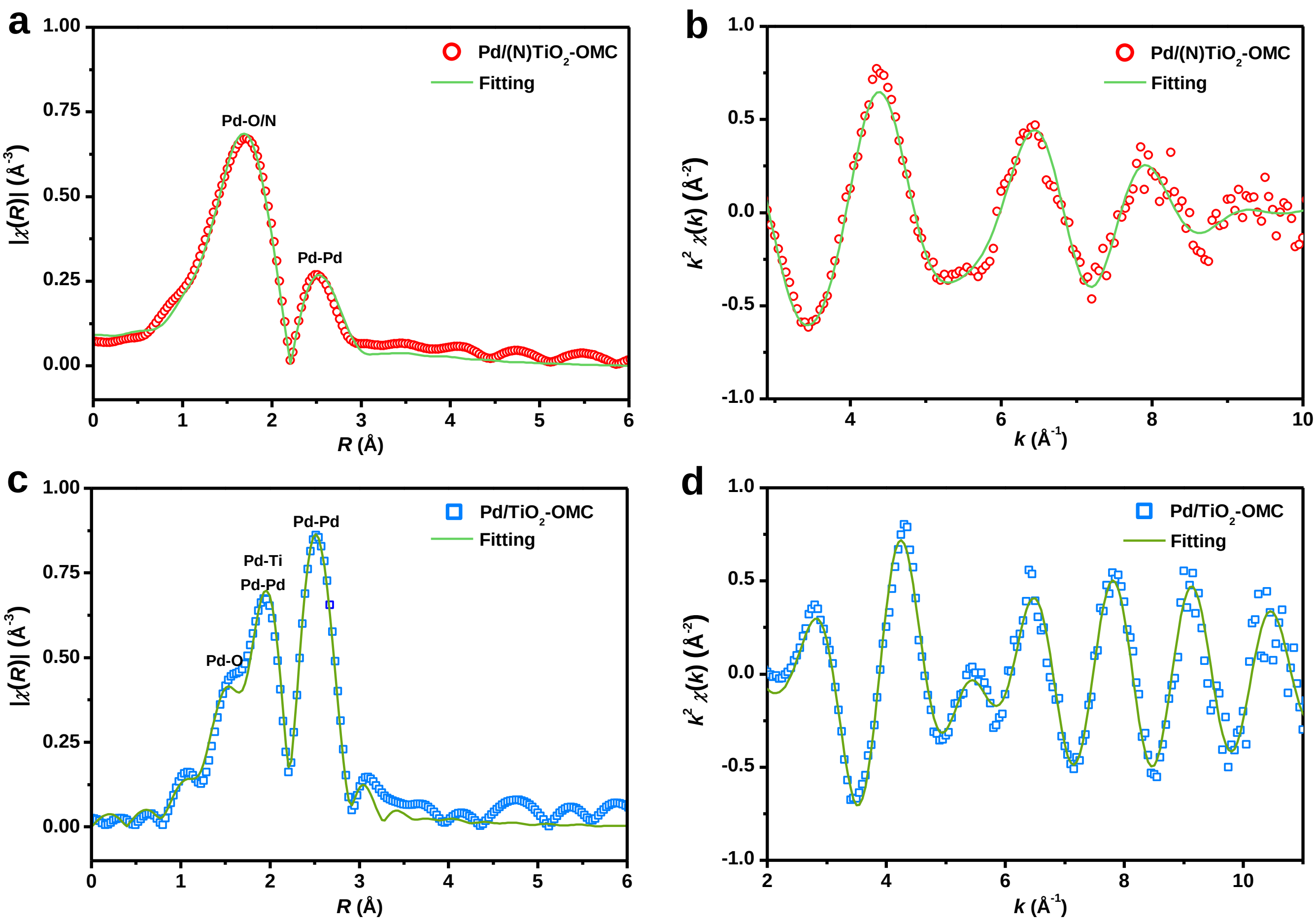

Supplementary Fig 10. Fitting results for X-ray absorption fine structure (XAFS) spectra. (**a, b**) The k^2-weighted and Fourier transformed magnitudes of the Pd K-edge EXAFS spectra for Pd/(N)TiO_2-OMC. (**c, d**) Pd K-edge EXAFS spectra and fitting result in k-space for Pd/(N)TiO_2-OMC and Pd/TiO_2-OMC. The plots are not corrected for phase shifts.

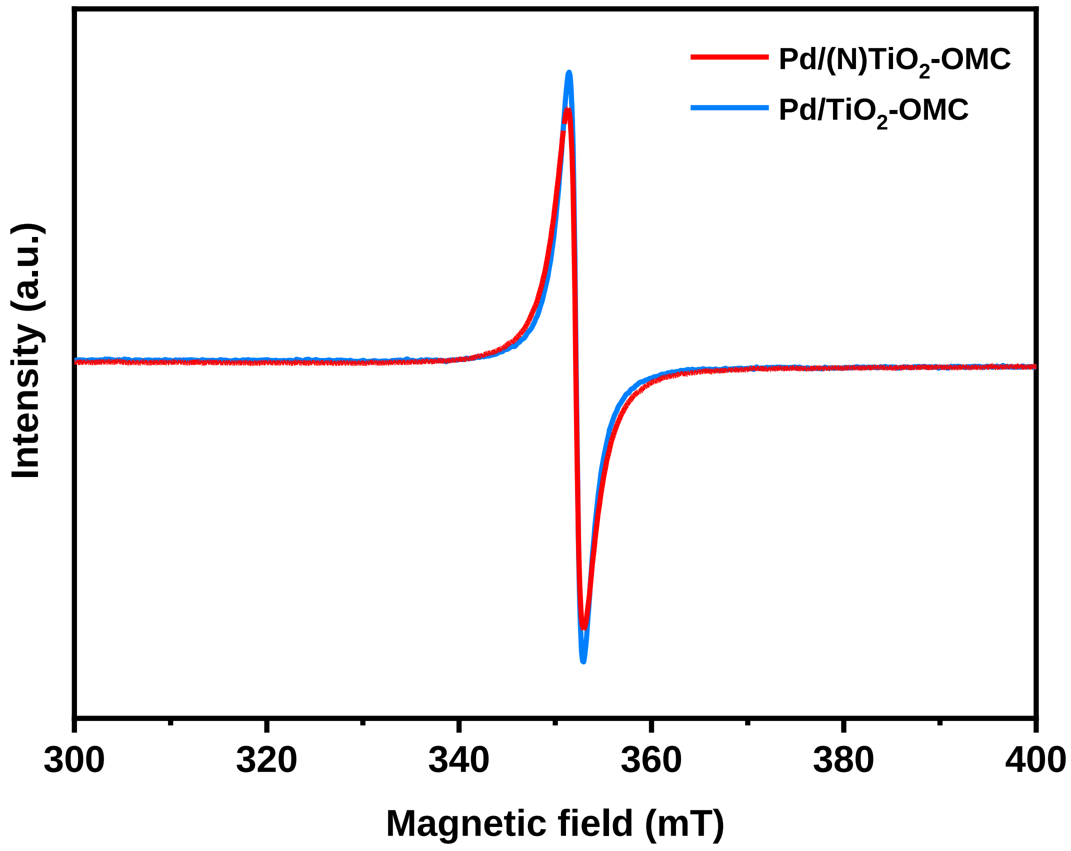

Supplementary Fig. 11. The presence of oxygen vacancy. The electron paramagnetic resonance (EPR) spectra of Pd/(N)TiO_2-OMC and Pd/TiO_2-OMC.

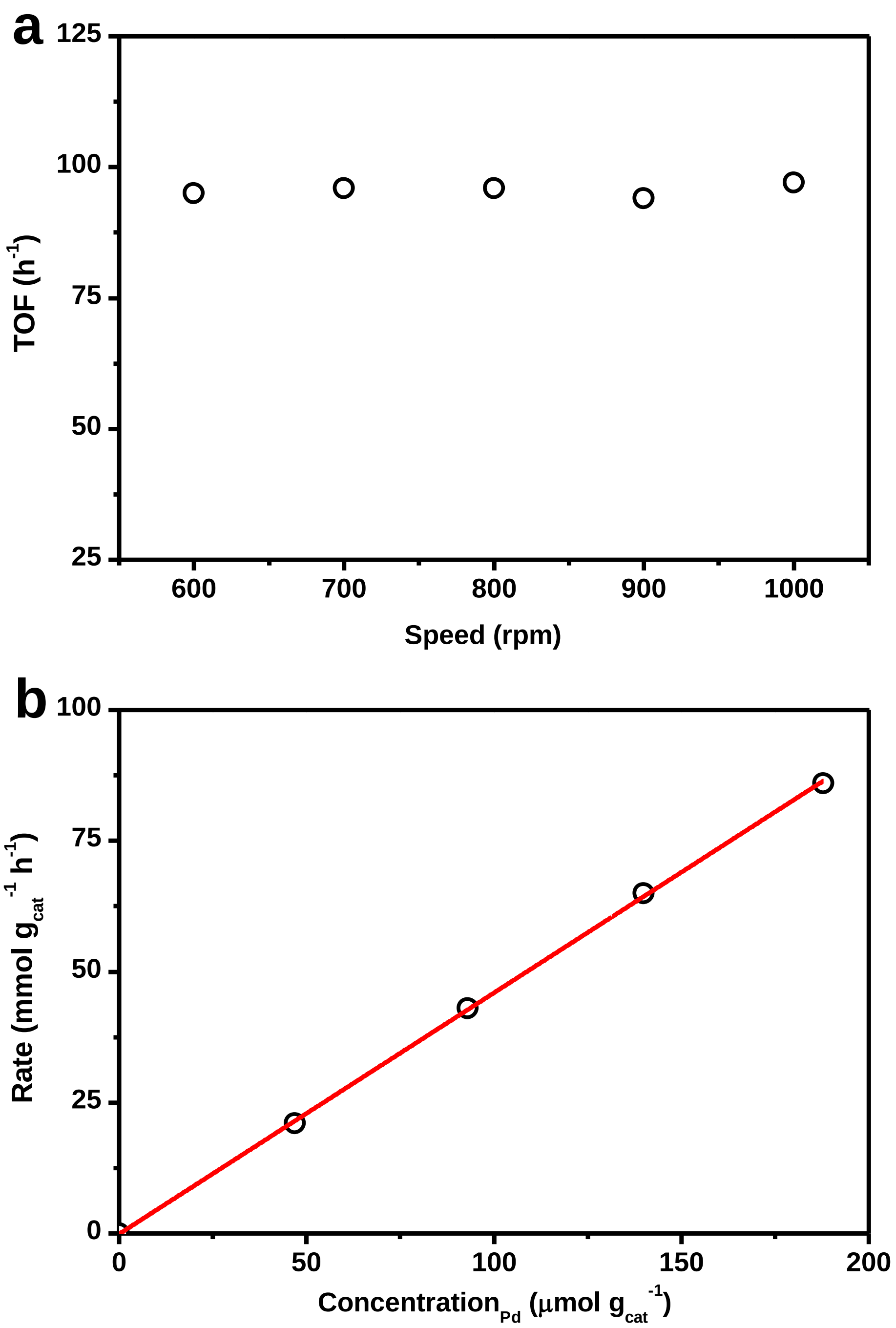

Supplementary Fig. 12. Mass transfer limitation test. (**a**) Effect of the stirring rate on the TOF of the direct methoxylation of 8-MeQ over the Pd/(N)TiO_2-OMC catalyst (Mass transfer limitation test). (**b**) Effect of Pd loading on the reaction rate of direct methoxylation of 8-MeQ using the Pd/(N)TiO_2-OMC catalyst (Madon-Boudart test). Reaction conditions: 21 mg of catalyst with a Pd concentration range of 0.5-2.0 *wt*%; 0.2 mmol 8-MeQ; 1.1 equivalent iodobenzene diacetate; 2 mL of methanol; 100 °C; atmospheric pressure; in air.

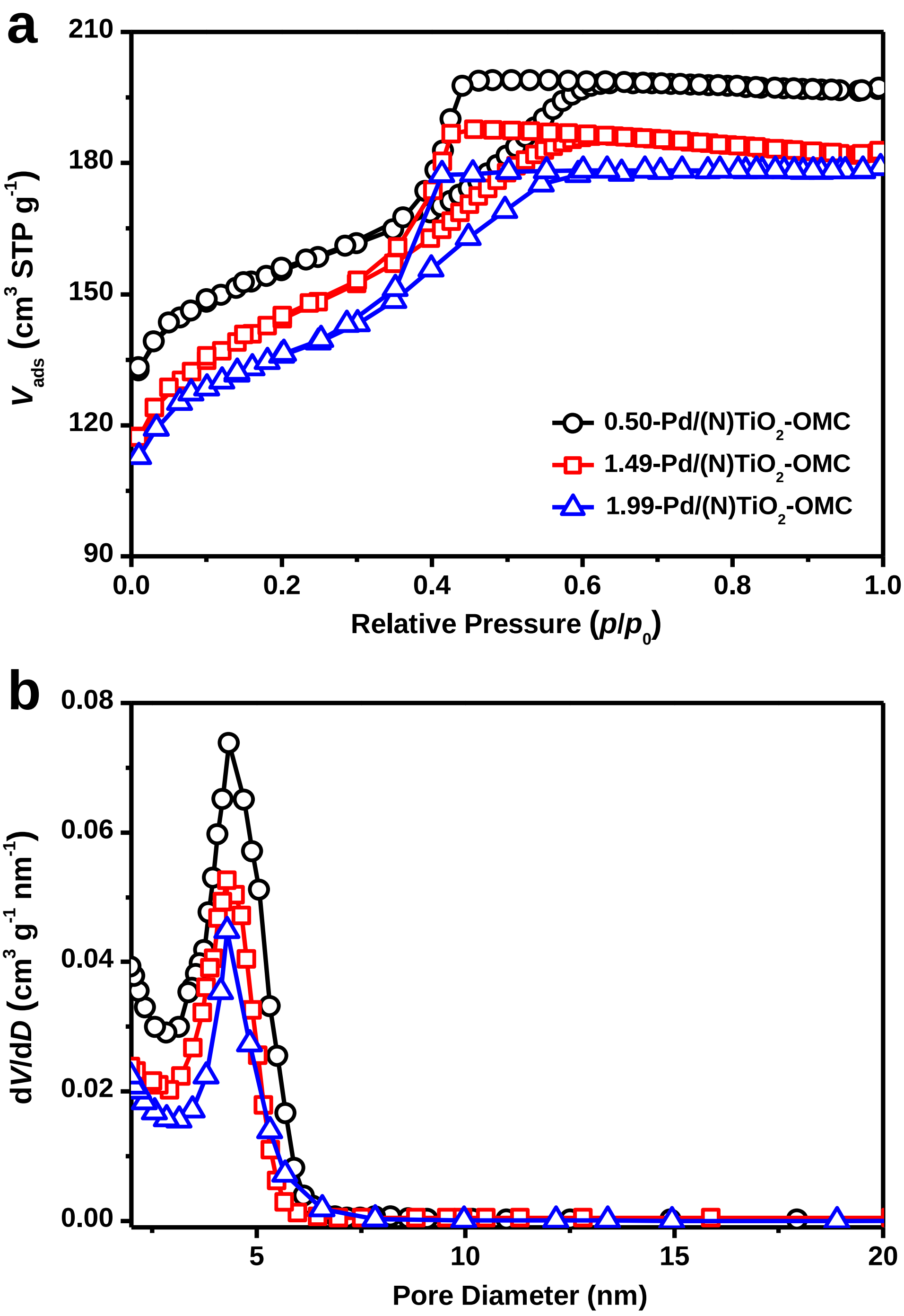

Supplementary Fig. 13. Pore structure for reference catalysts. (**a**) N_2 sorption isotherms and (**b**) pore size distribution curves for Pd/(N)TiO_2-OMC catalysts with Pd contents of 0.50, 1.49 and 1.99 *wt*%.

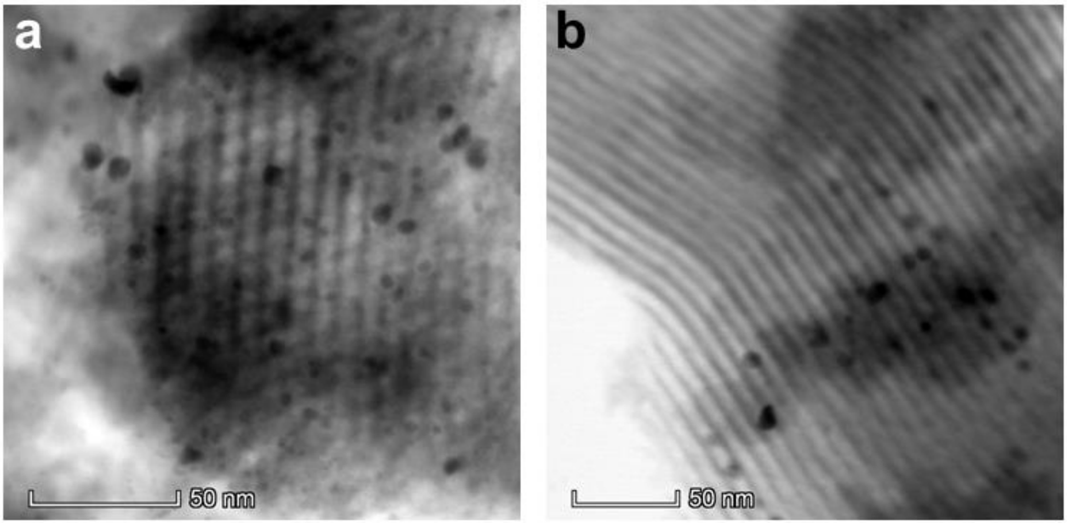

Supplementary Fig. 14. Structure of the reference catalyst. (**a, b**) TEM images of used Pd/SBA-15 catalysts.

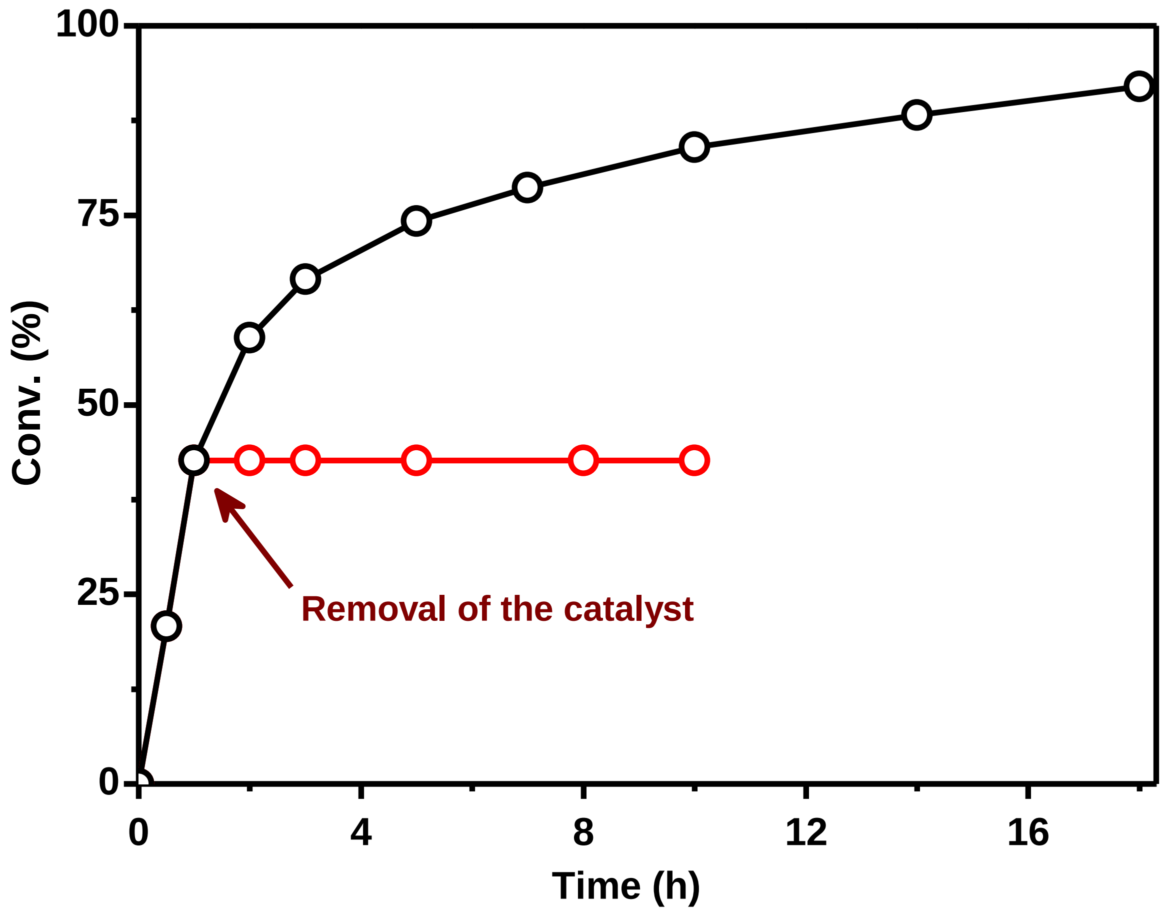

Supplementary Fig. 15. Hot filtration for Pd leaching test. The conversion plot for 8-MeQ over the Pd/(N)TiO_2-OMC catalyst in the hot filtration experiment (red open circle). The Pd/(N)TiO_2-OMC catalyst was removed by hot filtration after 1 h reaction at 100 ºC. A fresh substrate and $Ph_2I(OAc)_2$ were then added to the filtrate, while the reaction temperature was kept at 100 ºC. For comparison, the conversion plot for the 8-MeQ over a Pd nanocatalyst with reaction time is also provided (black open circle). Reaction conditions: 1 mol% Pd/(N)TiO_2-OMC catalyst; 0.2 mmol 8-MeQ; 1.1 equivalent iodobenzene diacetate; 2 mL of methanol; 100 ºC; atmospheric pressure; in air.

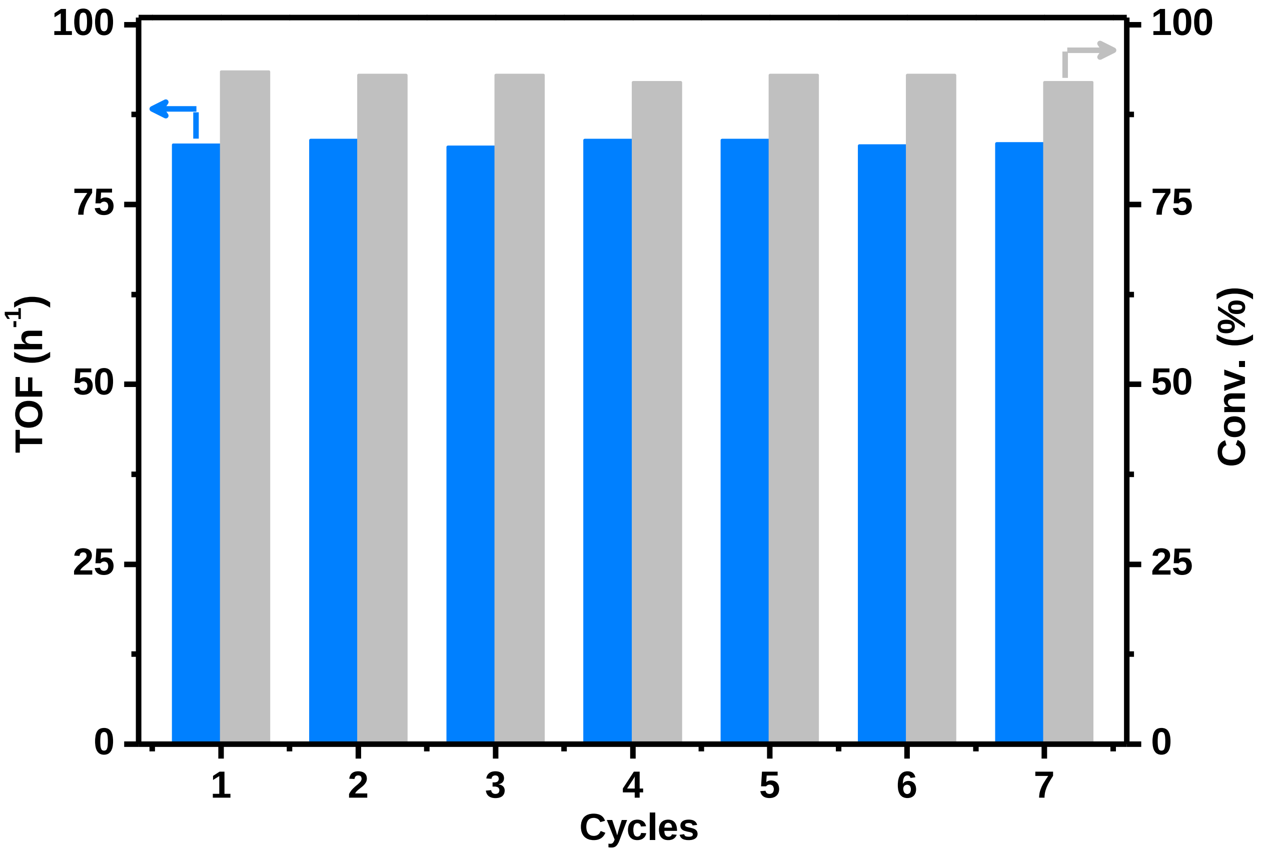

Supplementary Fig. 16. Reusability. Comparison of the TOF and conversion of 8-MeQ in successive runs over a recovered Pd/(N)TiO_2-OMC catalyst. Reaction conditions: 1 mol% Pd/(N)TiO_2-OMC catalyst; 0.2 mmol of 8-MeQ; 1.1 equivalent of iodobenzene diacetate; 2 mL of methanol; 100 ºC; 800 rpm; atmospheric pressure; in air.

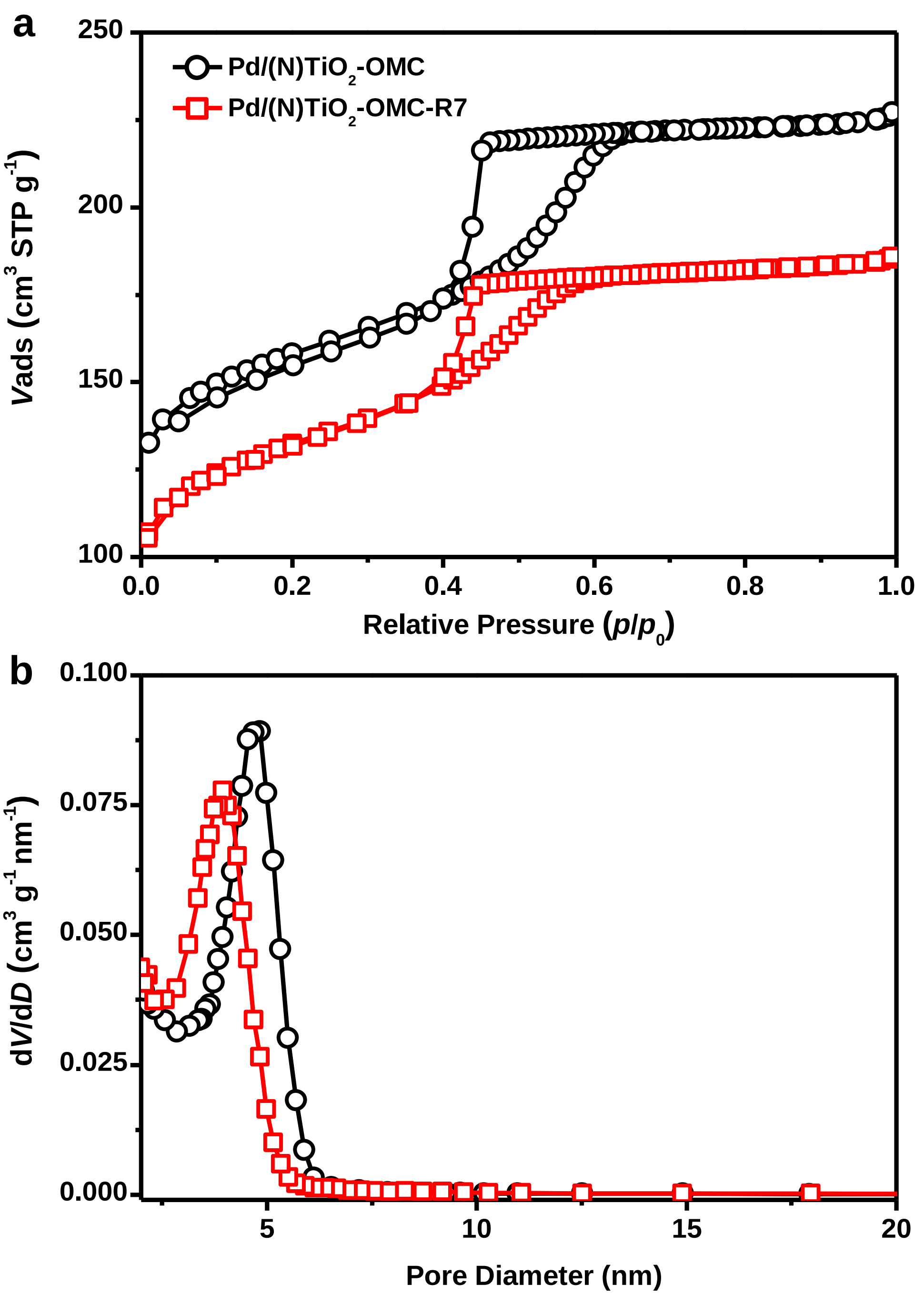

Supplementary Fig. 17. Pore structure stability. (a) N_2 sorption isotherms and (**b**) pore size distribution curves for fresh and used Pd/(N)TiO_2-OMC. Pd/(N)TiO_2-OMC-R7 is the catalyst after seven catalytic runs.

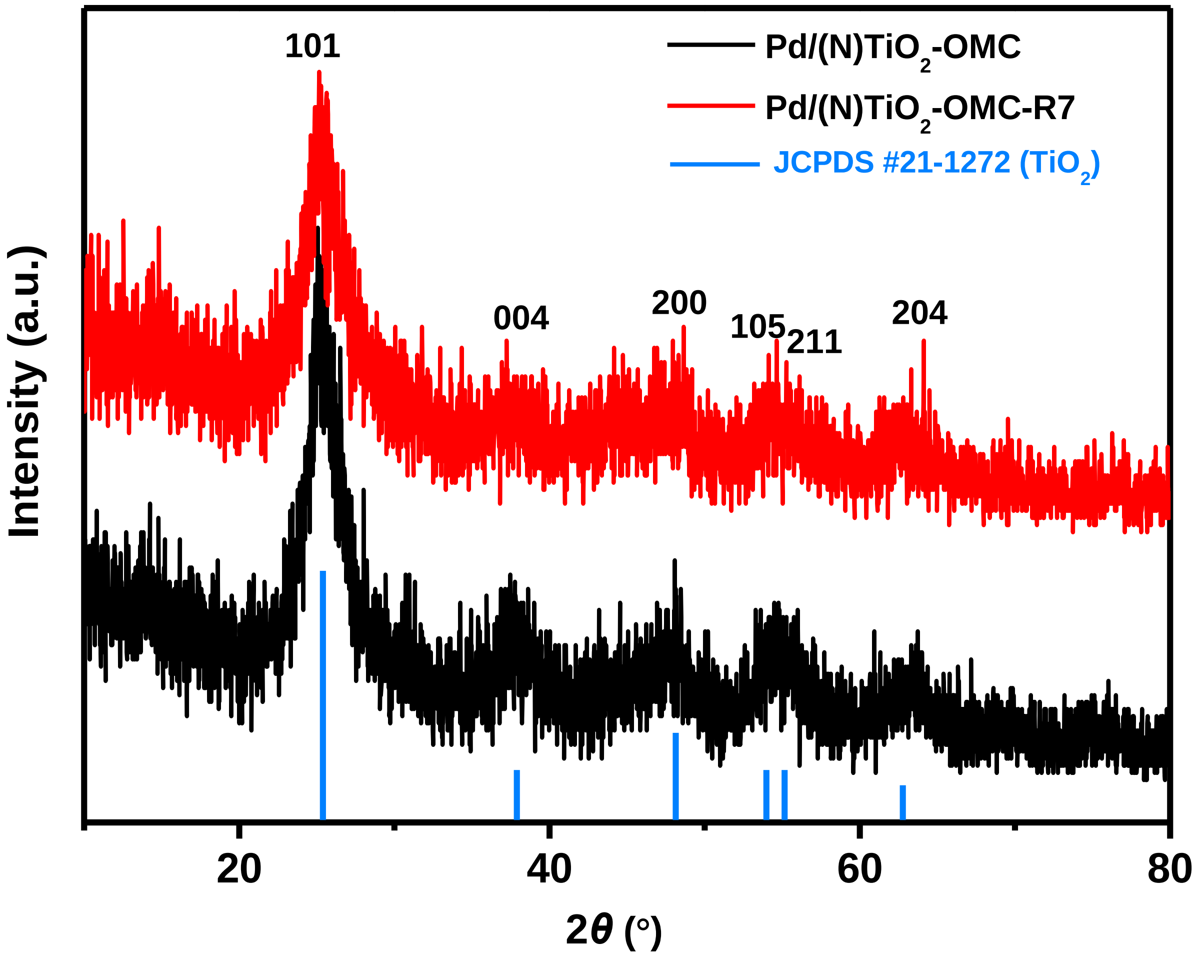

Supplementary Fig. 18. Structure and phase stability. Wide-angle X-ray diffraction patterns for fresh and used Pd/(N)TiO_2-OMC. Pd/(N)TiO_2-OMC-R7 is the catalyst after seven catalytic runs.

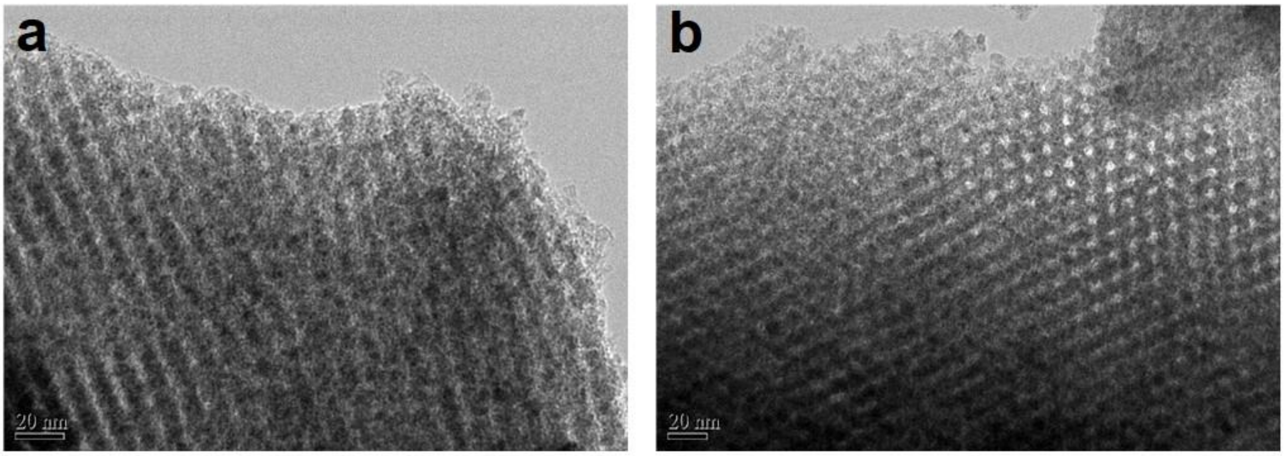

Supplementary Fig. 19. Structure and nanoparticle stability. (**a, b**) TEM images of the recycled Pd/(N)TiO_2-OMC-R7 after seven catalytic runs viewed along the [110] and [001] directions.

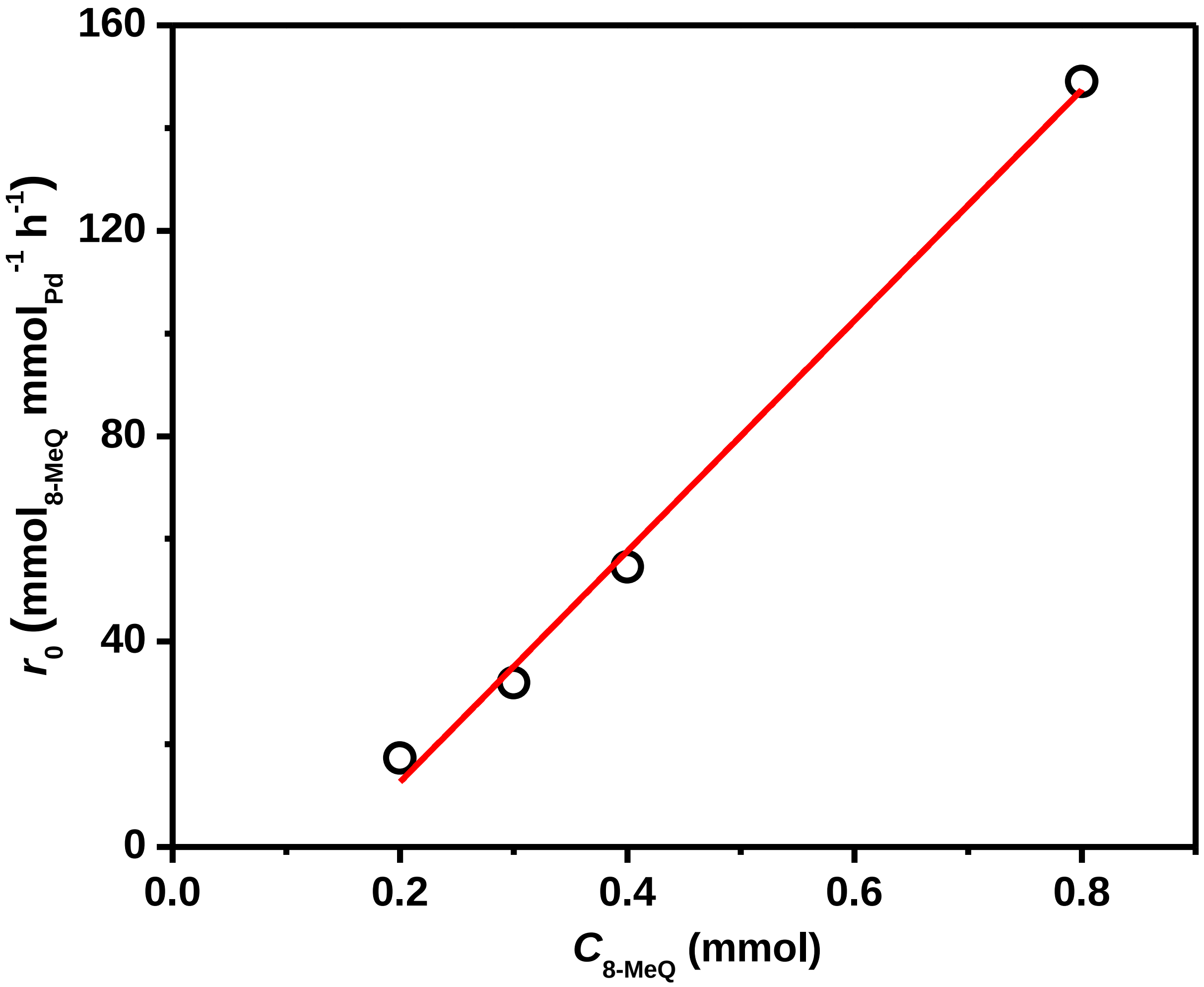

Supplementary Fig. 20. Kinetics study. Effect of 8-MeQ concentration on the reaction rate for the direct methoxylation of 8-MeQ over the Pd/(N)TiO_2-OMC catalyst. Reaction conditions: 1 mol% Pd/(N)TiO_2-OMC catalyst; 1.1 equivalent of iodobenzene diacetate; 2 mL of methanol; 100 ℃; 800 rpm; atmospheric pressure; in air.

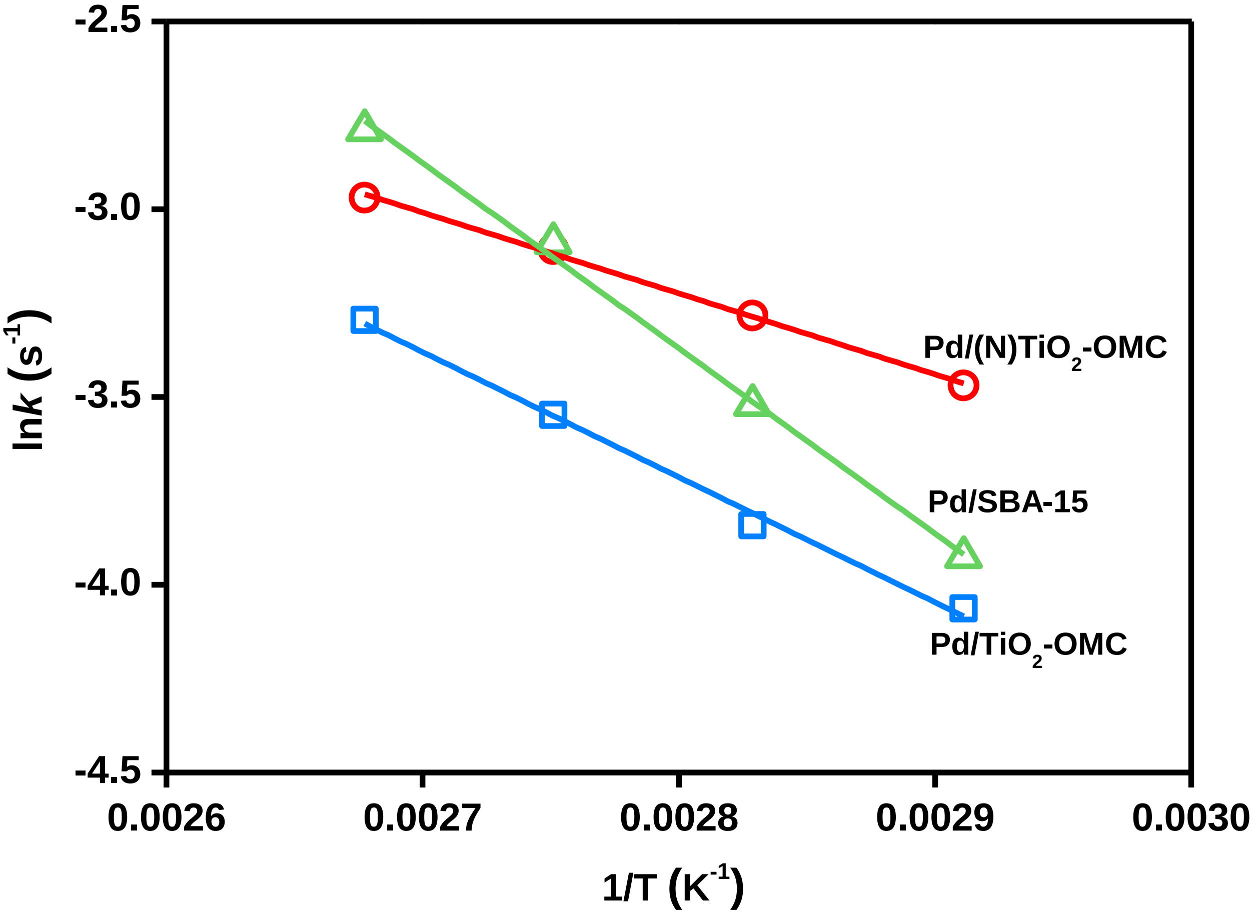

Supplementary Fig. 21. Arrhenius plot. Arrhenius plots for the direct methoxylation of 8-MeQ over the Pd/(N)TiO_2-OMC, Pd/TiO_2-OMC, and Pd/SBA-15 catalysts.

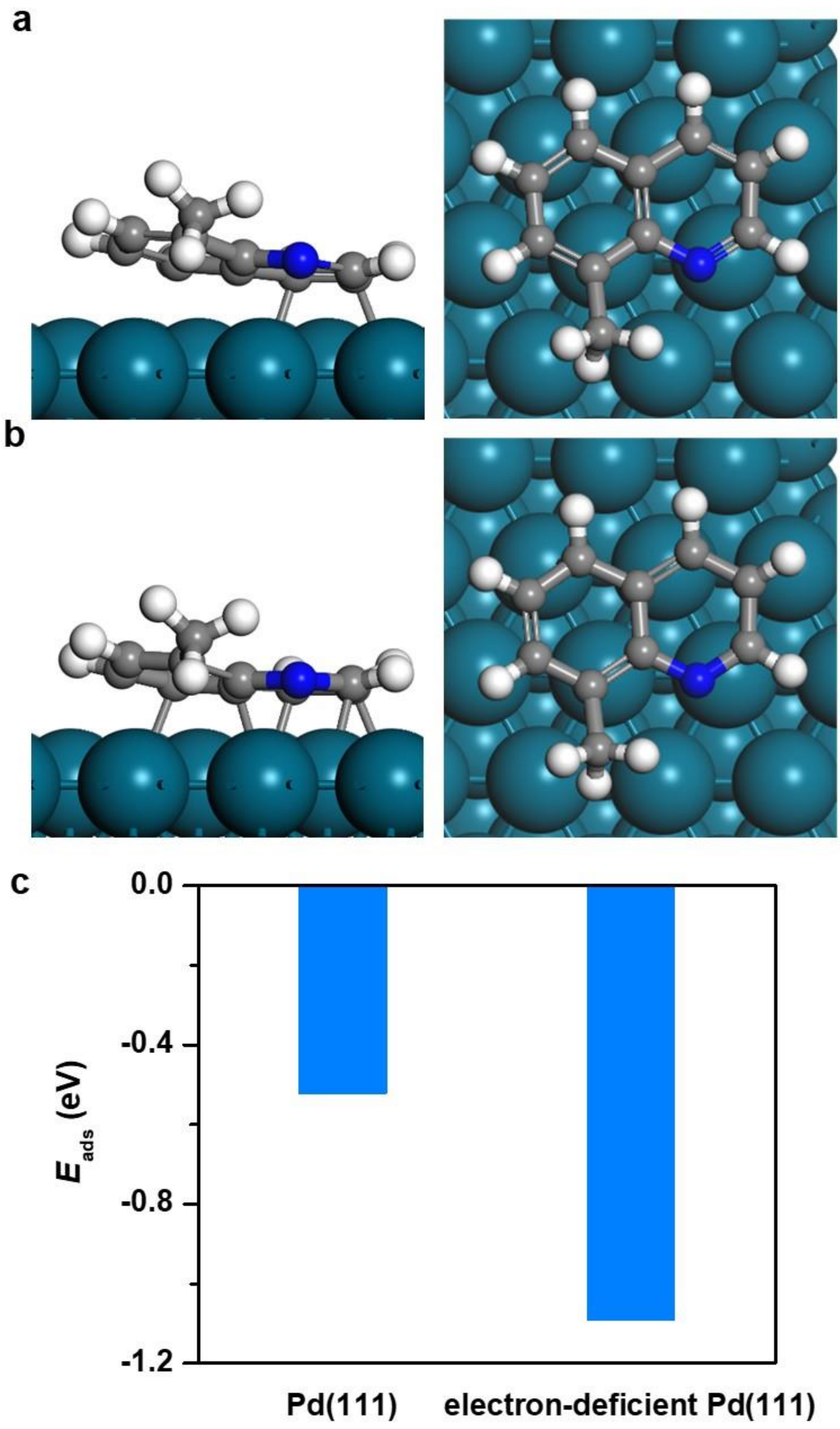

Supplementary Fig. 22. DFT calculation. Side and top views of the most stable adsorption configurations of 8-MeQ on Pd(111) (**a**) and electron-deficient Pd(111) (**b**), and the corresponding adsorption energies (**c**). Cyan, gray, white and blue balls represent Pd, C, H and N atoms, respectively.

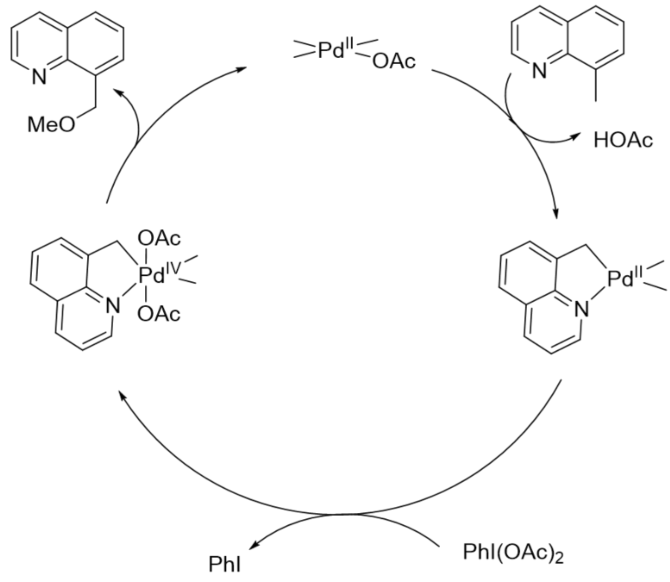

Supplementary Fig. 23. Possible homogeneous catalysis mechanism. The direct methoxylation of 8-MeQ by $Pd^{II/IV}$-involved catalysis[5,6].

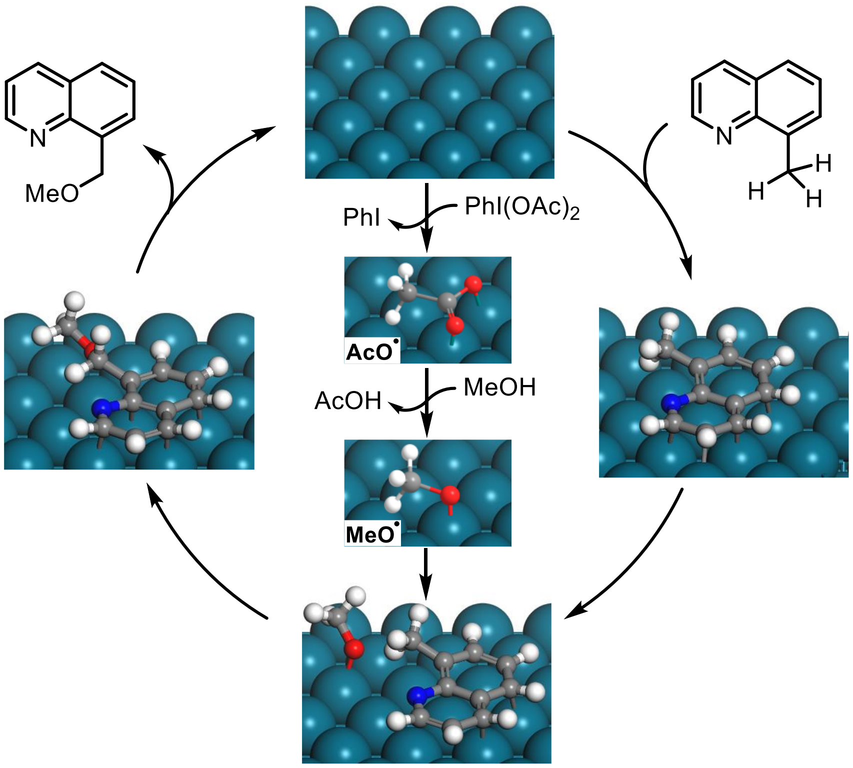

Supplementary Fig. 24. Possible heterogeneous catalysis mechanism. The direct methoxylation of 8-MeQ catalyzed over Pd/(N)TiO_2-OMC by a radical relay pathway. First, 8-MeQ adsorbs on the Pd surface to form *8-MeQ with an activation of the C(sp^3)–H bonds. At the same time, the adsorption of methanol on the electron-deficient Pd surface is facilitated, and the acetoxyl radical produced by the decomposition of $PhI(OAc)_2$ is transformed to form a methoxyl radical. The methoxyl radical species then react with the adjacent adsorbed *8-MeQ on the Pd surface with radical addition to the C(sp^3)–H and subtraction of a hydrogen atom. Finally, the adsorbed oxidative coupling product is desorbed from the Pd surface to regenerate available active Pd sites.

Supplementary Methods.

Synthesis of Pd/TiO_2-OMC. A Stöber solution was obtained by dissolving 1.1 mL of $TiCl_4$ in 1.0 g of distilled water and 10.0 g ethanol at 0 °C. A clear solution containing 1.5 g of F127, 1.0 g of deionized water and 8.0 g of ethanol was then mixed with the Stöber solution at 40 °C. After stirring the mixture for 30 min, a solution containing 5.0 g of 20 *wt*% low-polymerized phenolic resin was added. A further 1 h stirring yielded a claret colored mixture. The mixture was poured into multiple petri dishes that were immediately placed in an oven. After 24 h, the dishes were heated at 100 °C for further thermopolymerization of the carbonaceous resin. The resulting pale yellow and claret thin films were heated at 350 °C for 5 h to remove the template. Further crystallization of titania was accomplished at 550 °C for 1 h under a nitrogen atmosphere to obtain the TiO_2-OMC. Supported Pd catalysts were prepared by a wet impregnation method. In a typical procedure, 0.68 g of an aqueous solution of $PdCl_2$ (1.1 *wt*%) was mixed with 0.4 g of TiO_2-OMC carrier for 6 h under stirring. The mixture was evaporated, washed, and then dried. The washing solution was collected to determine the Pd concentration by ICP-AES. The washed solids were dried at 50 ºC under vacuum. The catalyst produced was reduced at 300 ºC in 10 *v*% H_2 in nitrogen for 3 h, and is referred to as the fresh supported catalyst (Pd/TiO_2-OMC).

Synthesis of Pd/SBA-15. Pristine mesoporous silica SBA-15 was synthesized by a hydrothermal method[7]. In a typical synthesis, 1.0 g of P123 block copolymer was dissolved with stirring in a solution of 7.5 g of water and 30.0 g of HCl (2 M), and then 2.08 g of TEOS was added with stirring at 40 ºC. After stirring for 20 h at 40 ºC, the gel solution was transferred into a Teflon bottle. The hydrothermal temperature and time were 100 ºC and 24 h, respectively. The SBA-15 carrier was obtained after removing the surfactant at 550 ºC under air. Supported Pd catalysts were prepared by the above wet impregnation method and are named Pd/SBA-15.

Synthesis of SH-SBA-15. SH-SBA-15 was synthesized by grafting mercaptopropyl groups onto pre-prepared mesoporous silica SBA-15[8]. A toluene (120 mL) suspension of SBA-15 (4.1 g) was then mixed with 10.0 g of 3-thiolpropyltrimethoxysilane at 110 ºC with reflux for 48 h; 1.8 mL of water was added to promote cross-linking, and the mixture was heated during reflux for an additional 24 h. The solids were then filtered and washed with copious amounts of toluene, hexane, and methanol to remove unreacted silanes. The recovered solids were Soxhlet extracted with dichloromethane at the reflux temperature for 24 h. The resulting white solids were collected, dried at room temperature overnight and then at 150 ºC for 3 h under vacuum. They were stored in a vacuum dryer.

Synthesis of low-polymerized phenolic resins. The carbon precursors (low molecular weight, soluble phenolic resins) were prepared from phenol and formaldehyde in a base-catalyzed process. In a typical procedure, 8.0 g of phenol was melted at 42 - 45 °C in a flask and mixed with 0.34 g of a 20 *wt*% aqueous sodium hydroxide (NaOH) solution under stirring. After 10 min, 5.24 g of formalin (37 *wt*% formaldehyde) was added. Then, the mixture was heated to 70 °C. After additional stirring for 1 h at this temperature, the mixture was cooled to room temperature. The pH value was adjusted to ~ 7.0 with a 2 M HCl solution. Water was removed by vacuum evaporation below 45 °C. The water- and ethanol-soluble phenolic resins were dissolved in ethanol (20 *wt*%) for further use.

EPR detection method. The capture of active radicals generated during the reaction was examined by EPR (Bruker ELEXSYS 500 spectrometer) using DMPO as a spin trapping agent. In general, a dried Schlenk tube equipped with a stir bar was loaded with 8-MeQ (0.2 mmol), methanol (2 mL) and catalyst (21 mg) at 100 °C. After 30 mins, DMPO (0.24 mmol) was immediately added to the filtered solution and transferred to a glass capillary tube. Then, the capillary tube was placed into a quartz EPR tube (φ4×250mm), and EPR spectra were recorded. Typical spectrometer parameters are shown as follows, scan range: 100 G; center field set: 3510 G; time constant: 1.25 ms; scan time: 40.96 s; modulation amplitude: 1.0 G; modulation frequency: 100 kHz; receiver gain: 1.00 × 103; microwave power: 19.17 mW. Signal fitting was carried out using the Spin Fit program (Bruker).

Theoretical calculations.

Spin-polarized first-principles calculations based on density functional theory (DFT) were performed using the Vienna Ab initio Simulation Package (VASP). The Perdew-Burke-Ernzerhof (PBE) exchange-correlation functional within the generalized gradient approximation (GGA) was used to describe the exchange-correlation energy. The projector-augmented-wave (PAW) method was used for the pseudopotentials. The energy cutoff for the plane wave basis expansion was set to 450 eV. The force on each atom was set as 0.03 eV/Å for the convergence criterion. Anatase(101) and Pd(111) were selected to construct the composite model. The different lattice parameters were matched by a resampling method. The surface model of Pd was constructed on the basis of the (111) lattice plane. All these had a vacuum layer of 20 Å in the z direction to avoid interaction between the layers. And a 5×6 super cell of a Pd(111) surface was used for the adsorption calculations. Sampling in the Brillouin zone was set by the Monkhorst-Pack method, the Monkhorst-Pack grid spacing was chosen to be greater than 0.06 $Å^{-1}$. The adsorption energy (E_{ads}) was calculated as:

$$E_{ads} = E_{total} – E_{molecule} – E_{surface} \quad (2)$$

where E_{total} represents the adsorbed molecule on the surface of Pd.

The free energies were calculated using the equation:

$$\Delta G = \Delta E_{DFT} + \Delta E_{ZPE} - T\Delta S \quad (3)$$

where ΔE_{DFT} is the DFT energy difference which is equal to "– E_{ads}" for the dissociation, and ΔE_{ZPE} and $T\Delta S$ are the zero-point energy correction and the change of entropy, respectively, which are obtained from vibration calculations.

X-ray absorption data analysis. The X-ray absorption fine structure (XAFS) spectra for the Pd *K*-edge data were obtained at the BL14W1 of the Shanghai Synchrotron Radiation Facility (SSRF). The samples were pressed into pellets and measured in the fluorescence mode. A double-crystal Si(311) monochromator was used for energy selection at the Pd *K*-edge (24,350 eV). The acquired XAFS data were processed according to standard procedures using the ATHENA module implemented in the IFEFFIT software packages[9]. The k^2-weighted EXAFS spectra were obtained by subtracting the post-edge background from the overall absorption and then normalizing with respect to the edge-jump step. Subsequently, k^2-weighted $\chi(k)$ data in

the k space ranging from 0 to 12.0 Å^{-1} were Fourier-transformed to real (R) space using a Hanning window (dk= 1.0 Å^{-1}) to separate the EXAFS contributions from the different coordination shells. The obtained amplitude reduction factor S_0^2 of a Pd foil was 0.77 and was fixed in the subsequent fitting of the Pd foil K-edge data for the catalyst. To obtain the quantitative structural parameters around the central atoms, least-squares curve parameter fitting was performed using the ARTEMIS module of the IFEFFIT software packages. The following EXAFS equation was used:

$$\chi(k) = \sum N_j F_j(k) \exp(-2\sigma_j^2 k_j^2) \sin(2krj + \phi_j(k)) / kr_j^2$$
$$k_j = (k^2 - 2m\Delta E_0 / h^2)^{1/2} \tag{4}$$

where N_j, r_j, σ_j, and ΔE_{0j} are the coordination number, the bond distance, the Debye-Waller factor, and the energy shift between reference and sample, respectively[10].

The XAFS spectra for the Pd L_3-edge data were obtained at the 4B7A station in the Beijing Synchrotron Radiation Facility (BSRF). A double-crystal Si(111) monochromator was used for energy selection at the Pd L_3-edge (3173 eV). The EXAFS spectra were obtained by subtracting the post-edge background from the overall absorption and then normalizing with respect to the edge-jump step. The difference in the number of 4d holes (d-charge gain) between the samples and Pd metal was evaluated from the Pd L-edges XANES using the following equation:

$$\Delta n_d = \frac{[(A_{\text{sample}} - A_{\text{Pd}})\ \text{Pd}\ L_3 + (A_{\text{sample}} - A_{\text{Pd}})\ \text{Pd}\ L_2]}{10.45} \tag{5}$$

where A is the peak area of the white lines at the L_3- or L_2-edges, and 10.45 is the absorption cross-section per hole in the 4d band of each Pd atom[11,12]. In order to eliminate the size effect, Pd/SBA-15 was used as the reference of the Pd metal. The ratio of L_3 peak area to L_2 peak area was determined to be about 2.5 according to the literature for simplification[13].

Kinetics calculations. The turn over frequency (TOF) for the Pd/(N)TiO_2-OMC catalyst was calculated on the basis of the estimated number of exposed palladium atoms, at a conversion below 20%.

$$\text{TOF}_{\text{Pd}} = \frac{n_{\text{Sub}} X}{n_{\text{Pd}} t \tau} \tag{6}$$

where n_{Sub} is the molar amount of the substrate, X is the conversion, n_{Pd} is the molar amount of Pd, t is the reaction time, and τ is the exposed surface atom dispersion. τ is measured by CO pulse adsorption.

Apparent activation energies (E_a) were calculated using the Arrhenius equation:

$$\ln k = \ln A - \frac{E_a}{RT} \tag{7}$$

where k is the reaction rate constant, A is the apparent pre-exponential factor, R is the universal gas constant and T is the reaction temperature. Taking into account the approximate first-order reaction kinetics for the direct methoxylation of 8-MeQ, k is simplified to be close to the initial reaction rate r_0.

The apparent enthalpy of activation (ΔS^{0*}) was determined as follows.

The TOF value was expressed in the Eyring form[14,15]:

$$\mathrm{TOF_{Pd}} = \frac{k_\mathrm{B}T}{h}\exp(\frac{\Delta S^{0*}}{R})\exp(-\frac{\Delta H^{0*}}{RT}) \quad (8)$$

where k_B, h, ΔS^{0*}, and ΔH^{0*} is the Boltzmann constant, Planck constant, entropy of activation, and enthalpy of activation, respectively.

The apparent activation energy E_a was related to ΔH^{0*} by the Temkin equation:

$$E_a = \Delta H^{0*} + \sum n_i \Delta H_i \quad (9)$$

where ΔH_i and n_i is the adsorption enthalpy and the reaction order of reactant i, respectively.

The entropy change in the activation step of the chemical reaction was closely related to the thermodynamics of the rate constant, which results in the following equation:

$$\Delta S^{0*} = R\ln(\frac{Ah}{k_\mathrm{B}Te\tau}) \quad (10)$$

^{1}H NMR Data.

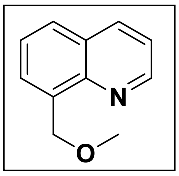

8-(methoxymethyl)-quinoline. Pd/(N)TiO_2-OMC (1 mol% of Pd), 8-MeQ (0.2 mmol), $PhI(OAc)_2$ (0.22 mmol), and MeOH (2.0 mL) were added to a 10 mL vial. The solution was heated with stirring to 100 °C for 18 hours. The product was obtained as a white solid (92% yield) after purification by column chromatography. R_f = 0.20 (hexanes/ethyl acetate 80/20) **^{1}H NMR (400 MHz, $CDCl_3$, ppm)** δ 8.84 (dd, *J* = 4.0 Hz, 2.0 Hz, 1H), 8.05 (dd, *J* = 8.0 Hz, 2.0 Hz, 1H), 7.74 (dd, *J* = 8.0 Hz, 2.0 Hz, 1H), 7.65 (d, *J* = 8 Hz, 1H), 7.45 (t, *J* = 7.8 Hz, 1H), 7.31 (dd, *J* = 8.0 Hz, 4.0 Hz, 1H), 5.12 (s, 2H), 3.50 (s, 3H).

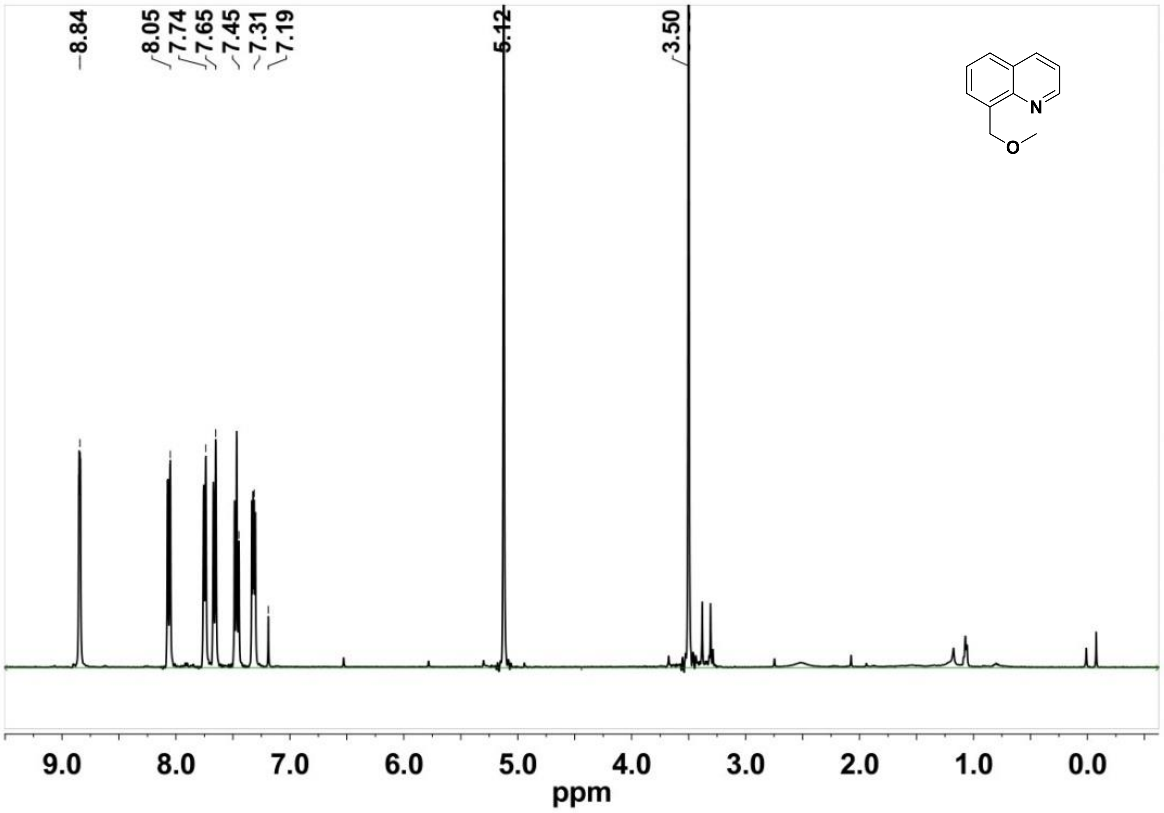

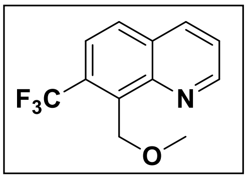

7-(Trifluoromethyl)-8-(methoxymethyl)-quinoline. Pd/(N)TiO_2-OMC (1 mol% of Pd), 7-(trifluoromethyl)-8-methylquinoline (0.2 mmol), $PhI(OAc)_2$ (0.22 mmol), and MeOH (2.0 mL) were added to a 10 mL vial. The solution was heated with stirring to 100 °C for 18 hours. The product was obtained as a light yellow oil (81% yield) after purification by column chromatography (hexanes/ethyl acetate = 80/20) **^{1}H NMR (400 MHz, $CDCl_3$, ppm)** δ 8.98 – 9.00 (dd, *J* = 8.0 Hz, 4.0 Hz, 1H), 8.06 – 8.08 (dd, *J* = 8.0 Hz, 2.0 Hz, 1H), 7.78 – 7.80 (d, *J* = 8.0 Hz, 1H), 7.69 – 7.71 (d, *J* = 8.0 Hz, 1H), 7.39 – 7.42 (dd, *J* = 8.0 Hz, 4.0 Hz, 1H), 5.22 (s, 2H), 3.47 (s, 3H).

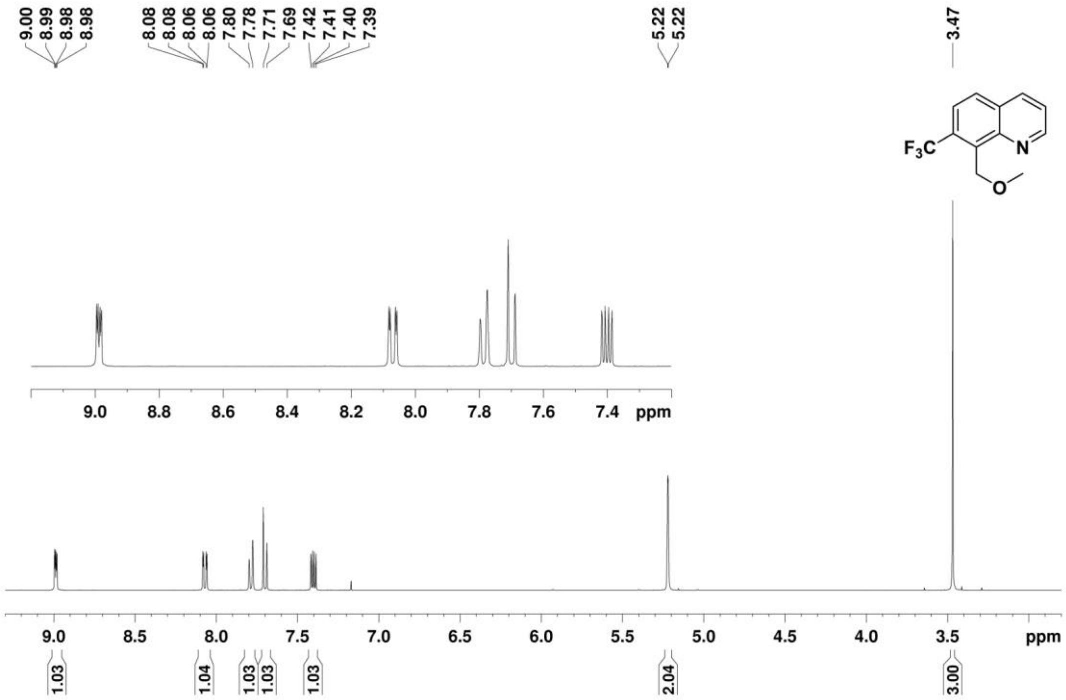

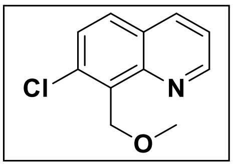

7-Chloro-8-(methoxymethyl)-quinoline. Pd/(N)TiO_2-OMC (1 mol% of Pd), 7-Chloro-8-methylquinoline (0.2 mmol), $PhI(OAc)_2$ (0.22 mmol), and MeOH (2.0 mL) were added to a 10 mL vial. The solution was heated with stirring to 100 °C for 18 hours. The product was obtained as a light color oil (73% yield) after purification by column chromatography (hexanes/ethyl acetate = 90/10). **^{1}H NMR (400 MHz, $CDCl_3$, ppm)** δ 8.99 – 9.01 (dd, *J* = 8.0 Hz, 4.0 Hz, 1H), 8.11 – 8.14 (dd, *J* = 8.4 Hz, 4.0 Hz, 1H), 7.73 – 7.75 (d, *J* = 8.0 Hz, 1H), 7.56 – 7.58 (d, *J* = 8.0 Hz, 1H), 7.40 – 7.43 (dd, *J* = 8.0 Hz, 4.2 Hz, 1H), 5.30 (s, 2H), 3.53 (s, 3H).

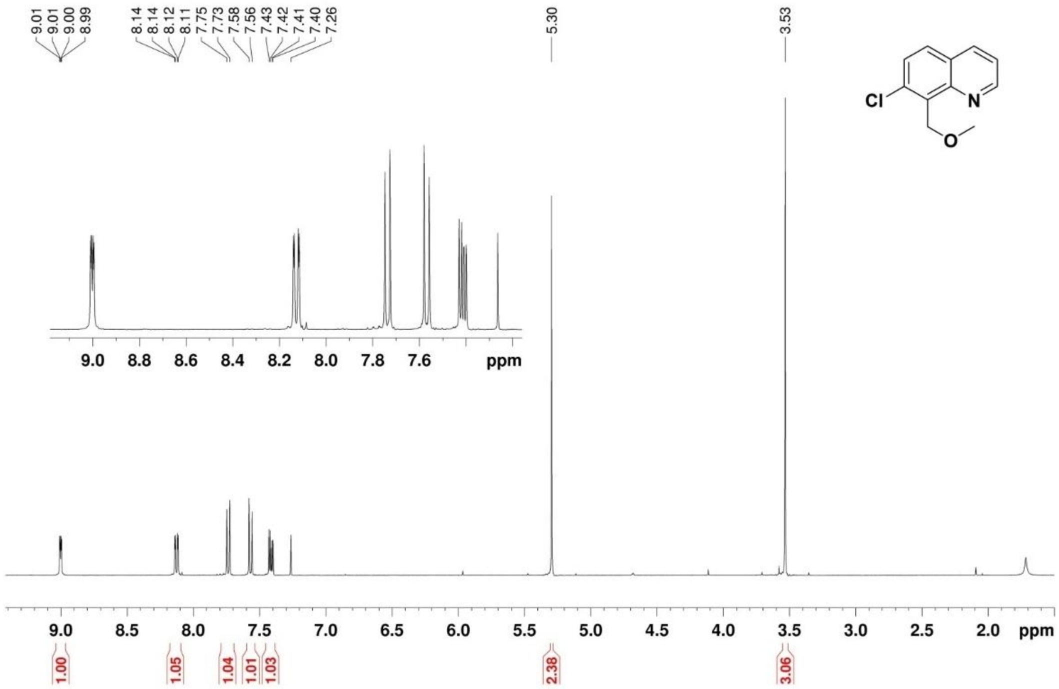

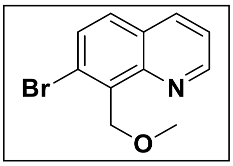

7-Bromo-8-(methoxymethyl)-quinoline. Pd/(N)TiO_2-OMC (1 mol% of Pd), 7-bromo-8-MeQ (0.2 mmol), $PhI(OAc)_2$ (0.22 mmol), and MeOH (2.0 mL) were added to a 10 mL vial. The solution was heated with stirring to 100 °C for 18 hours. The product was obtained as a yellow oil (84% yield) after purification by column chromatography. **^{1}H NMR (400 MHz, $CDCl_3$, ppm)** δ 8.98 – 9.00 (dd, *J* = 8.0 Hz, 2.0 Hz, 1H), 8.10 – 8.13 (dd, *J* = 8.0 Hz, 4.0 Hz, 1H), 7.72 – 7.74 (d, *J* = 8.0 Hz, 1H), 7.63 – 7.66 (d, *J* = 12.0 Hz, 1H), 7.41 – 7.44 (dd, *J* = 8.4 Hz, 4.2 Hz, 1H), 5.31 (s, 2H), 3.53 (s, 3H).

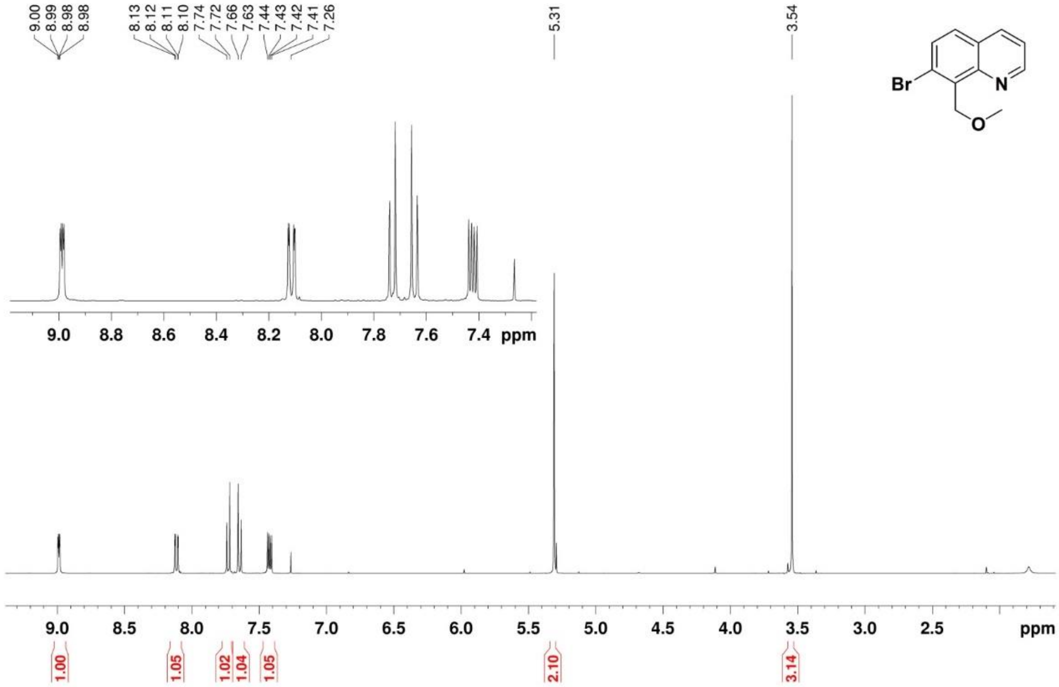

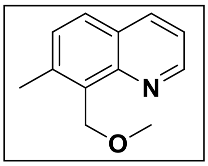

7-Methyl-8-(methoxymethyl)-quinoline. Pd/(N)TiO_2-OMC (1 mol% of Pd), 7- methyl-8-methylquinoline (0.2 mmol), $PhI(OAc)_2$ (0.22 mmol), and MeOH (2.0 mL) were added to a 10 mL vial. The solution was heated with stirring to 100 °C for 18 hours. The product was obtained as a light yellow oil (86% yield) after purification by column chromatography (hexanes/ethyl acetate = 80/20). **^{1}H NMR (400 MHz, $CDCl_3$, ppm)** δ 8.84 – 8.85 (dd, *J* = 4.0 Hz, 2.0 Hz, 1H), 7.97 – 7.99 (dd, *J* = 8.0 Hz, 2.0 Hz, 1H), 7.58 – 7.60 (d, *J* = 8.0 Hz, 2H), 7.30 – 7.32 (d, *J* = 8.0 Hz, 2H), 7.21 – 7.24 (dd, *J* = 8.2 Hz, 4.0 Hz, 1H), 5.16 (s, 2H), 3.41 (s, 3H), 2.55 (s, 3H).

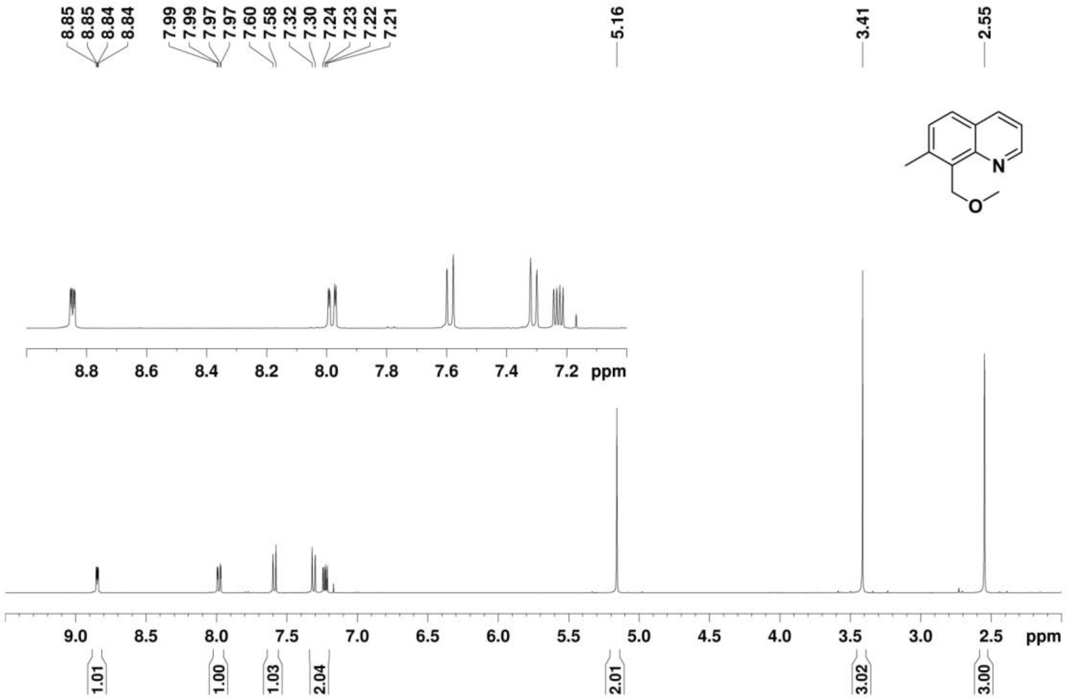

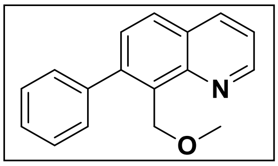

7-Phenyl-8-(methoxymethyl)-quinoline. Pd/(N)TiO_2-OMC (1 mol% of Pd), 7-phenyl-8-methylquinoline (0.2 mmol), $PhI(OAc)_2$ (0.22 mmol), and MeOH (2.0 mL) were added to a 10 mL vial. The solution was heated with stirring to 100 °C for 18 hours. The product was obtained as a light colored solid (85% yield) after purification by column chromatography (hexanes/ethyl acetate = 80/20). **^{1}H NMR (400 MHz, $CDCl_3$, ppm)** δ 9.04 – 9.05 (t, *J* = 4.0 Hz, 1H), 8.13 – 8.15 (d, *J* = 8.0 Hz, 1H), 7.80 – 7.82 (d, *J* = 8.0 Hz, 2H), 7.53 – 7.59 (dd, *J* = 12.0 Hz, 8.0 Hz, 1H), 7.46 – 7.50 (m, 3H), 7.41 – 7.43 (m, 2H), 7.38 – 7.40 (m, 2H), 4.95 (s, 2H), 3.48 (s, 3H).

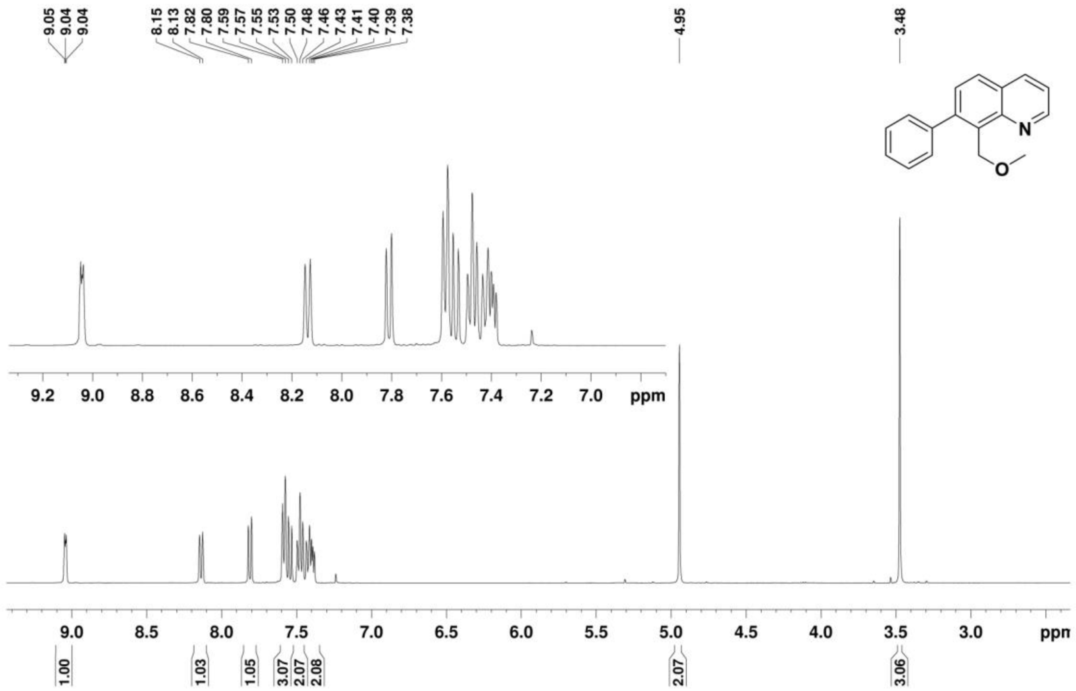

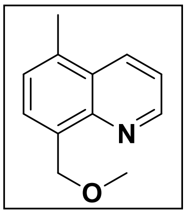

7-Methyl-8-(methoxymethyl)-quinoline. Pd/(N)TiO_2-OMC (1 mol% of Pd), 7,8-dimethylquinoline (0.2 mmol), $PhI(OAc)_2$ (0.22 mmol), and MeOH (2.0 mL) were added to a 10 mL vial. The solution was heated with stirring to 100 °C for 18 hours. The product was obtained as a light yellow solid (82% yield) after purification by column chromatography (hexanes/ethyl acetate = 80/20). **^{1}H NMR (400 MHz, $CDCl_3$, ppm)** δ 8.90 – 8.92 (dd, *J* = 4.0 Hz, 2.0 Hz, 1H), 8.26 – 8.29 (dd, *J* = 8.0 Hz, 4.0 Hz, 1H), 7.67 – 7.69 (d, *J* = 8.0 Hz, 1H), 7.38 – 7.41 (dd, *J* = 8.0 Hz, 4.2 Hz, 1H), 7.34 – 7.35 (d, *J* = 4.0 Hz, 3H), 5.15 (s, 2H), 3.55 (s, 3H), 2.64 (s, 3H).

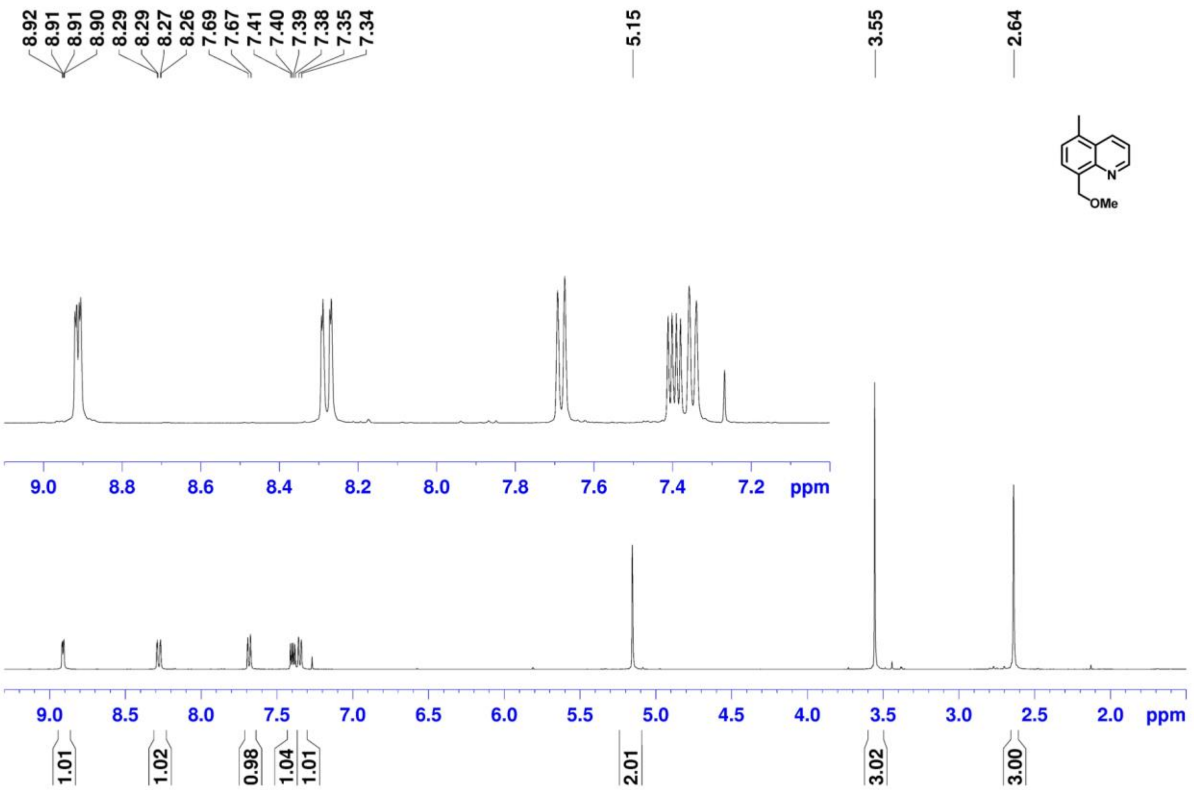

Supplementary References

1 Henkelman, G., Arnaldsson, A. & Jónsson, H. A fast and robust algorithm for Bader decomposition of charge density. *Comp. Mater. Sci.* **36**, 354-360 (2006).

2 Yang, S.-D. et al. Palladium-catalyzed direct arylation of (hetero)arenes with aryl boronic acids. *Angew. Chem. Int. Ed.* **47**, 1473-1476 (2008).

3 Ehlers, P. et al. Synthesis of 2,5-diarylpyrroles by ligand-free palladium-catalyzed C-H activation of pyrroles in ionic liquids. *ChemCatChem* **5**, 2504-2511 (2013).

4 Deprez, N. R., Kalyani, D., Krause, A. & Sanford, M. S. Room temperature palladium-catalyzed 2-arylation of indoles. *J. Am. Chem. Soc.* **128**, 4972-4973 (2006).

5 Dick, A. R., Kampf, J. W. & Sanford, M. S. Unusually stable palladium(IV) complexes: detailed mechanistic investigation of C−O bond-forming reductive elimination. *J. Am. Chem. Soc.* **127**, 12790-12791 (2005).

6 Lyons, T. W. & Sanford, M. S. Palladium-catalyzed ligand-directed C−H functionalization reactions. *Chem. Rev.* **110**, 1147-1169 (2010).

7 Duan, L. L. et al. An efficient reusable mesoporous solid-based Pd catalyst for selective C2 arylation of indoles in water. *ACS Catal.* **6**, 1062–1074 (2016).

8 Sakamoto, Y. et al. Synthesis of platinum nanowires in organic−inorganic mesoporous silica templates by photoreduction: formation mechanism and isolation. *J. Phys. Chem. B* **108**, 853-858 (2004).

9 Ravel, B. & Newville, M. ATHENA, ARTEMIS, HEPHAESTUS: data analysis for X-ray absorption spectroscopy using IFEFFIT. *J. Synchrotron. Radi.* **12**, 537-541 (2005).

10 Inoue, T., Asakura, K. & Iwasawa, Y. Characterization of Pt/SbO_x catalysts active for selective oxidation of isobutane by means of XRD, TEM, and XAFS. *J. Catal.* **171**, 457-466 (1997).

11 Gatla, S. et al. Influence of Sb on the structure and performance of Pd-based catalysts: an X-ray spectroscopic study. *J. Phys. Chem. C* **121**, 3854–3861 (2017).

12 Starace, A. F. Potential-barrier effects in photoabsorption. I. General theory. *Phys. Rev. B* **5**, 1773 (1972).

13 Sham, T. K. *L*-edge X-ray-absorption spectra of $PdAl_3$ and $PdCl_2$: a study of charge redistribution in compounds of an element with a nearly full 4*d* shell. *Phys. Rev. B* **31**, 1903-1908 (1985).

14 Chen, W. et al. Mechanistic and kinetic insights into the Pt-Ru synergy during hydrogen generation from ammonia borane over PtRu/CNT nanocatalysts. *J. Catal.* **356**, 186-196 (2017).

15 Teschner, D. et al. In situ surface coverage analysis of RuO_2-catalysed HCl oxidation reveals the entropic origin of compensation in heterogeneous catalysis. *Nat. Chem.* **4**, 739-745 (2012).
